# Predicting the Clinical Outcome of Lung Adenocarcinoma Using a Novel Gene Pair Signature Related to RNA-Binding Protein

**DOI:** 10.1155/2020/8896511

**Published:** 2020-10-26

**Authors:** Liangliang Meng, Xiaoxi He, Xiao Zhang, Xiaobo Zhang, Yingtian Wei, Bin Wu, Jing Li, Yueyong Xiao

**Affiliations:** ^1^Medical School of Chinese PLA, Beijing, China; ^2^Department of Radiology, The First Medical Centre, Chinese PLA General Hospital, Beijing, China; ^3^Department of Radiology, Chinese PAP Beijing Corps Hospital, Beijing, China; ^4^Department of Radiology, Tianjin Medical University Cancer Institute and Hospital, National Clinical Research Center for Cancer, Tianjin, China

## Abstract

Adenocarcinoma is the most common type of lung cancer, and patients have varying prognoses. RNA-binding proteins (RBP) are deemed to be closely associated with tumorigenesis and development, but the exact mechanism is currently unknown. This study was aimed at constructing a new robust prognostic model based on RNA-binding protein-related gene pair scores for better clinical guidance. The model for this study was constructed based on data of lung adenocarcinoma from The Cancer Genome Atlas (TCGA) database. Prognosis-related RBP gene pair models were created based on differentially expressed genes, and the accuracy of the models was verified in a different age, staging, and other subdatasets. A total of 379 RNA-binding protein-related genes were differentially expressed in tumor tissue. From these genes, we constructed a prognostic model consisting of 33 gene pairs, which were found to be significantly associated with survival in TCGA dataset (*P* < 0.0001, hazard ratio (HR) = 4.380 (3.139 to 6.111)) and different subdatasets. As expected, the results were verified in the GEO validation cohort (*P* = 7.8 × 10^−3^, HR = 1.597 (1.095 to 2.325)). We found that the signature exhibited an independent prognostic factor in both the univariate and multivariate Cox regression analyses (*P* < 0.001). CIBERSORT was applied to estimate the fractions of infiltrated immune cells in bulk tumor tissues. CD8 T cells, activated dendritic cells, regulatory T cells (Tregs), and activated CD4 memory T cells presented a significantly lower fraction in the high-risk group (*P* < 0.01). Patients in the high-risk group had significantly higher tumor mutational burden (TMB) (*P* = 4.953*e* − 04) and lower levels of immune cells (*P* = 3.473*e* − 05) and stromal cells (*P* = 0.005) in the tumor microenvironment than those in the low-risk group. Furthermore, the Protein-protein interaction (PPI) network and various enrichment analyses have genuinely uncovered the interrelationships and potential functions of the RBP genes within the model. The results of the present study validated the importance of RNA-binding proteins in tumorigenesis and progression and support the RBP gene-related signature as a promising marker for prognosis prediction in lung adenocarcinoma.

## 1. Introduction

Non-small-cell lung cancer typically includes lung adenocarcinoma and lung squamous carcinoma, with lung adenocarcinoma accounting for about 40-55% of all lung cancer patients [1]. Unlike lung squamous carcinoma, lung adenocarcinomas tend to originate in the smaller bronchial tubes and are therefore more likely to occur in the peripheral lobe of the lung and are more likely to occur in women and nonsmokers. The prognosis of patients with lung adenocarcinoma is usually closely related to the pathological type of the tumor, the pathological stage, the genetic characteristics of the patients, and the selectivity of surgery or targeted therapy options [1, 2]. Overall, patients with larger inoperable tumors or early metastases usually have a poorer prognosis and reduced survival. For earlier, more localized lung adenocarcinoma lesions, surgery has been the preferred option with excellent results [3]. In inoperable patients, a puncture biopsy of the lesion, genetic testing, and immunohistochemical testing are usually performed clinically to determine whether molecular targeted therapy or immunotherapy can be used properly [4]. Commonly used targeted therapeutic agents primarily target tumor angiogenesis and epidermal growth factor receptors. Immune checkpoint inhibitors targeting the PD-1/PD-L1 pathway have shown significant efficacy in the treatment of some patients [5]. Patients with poor immunotherapy or targeted therapy will require systemic chemotherapy and chemotherapy.

RNA-binding protein (RBP) is a category of proteins that accompany RNA to regulate the metabolic process and bind to RNA. Approximately 60% of RNA-binding proteins were found to interact extensively with chromatin and are enriched in gene promoter and enhancer regions [6]. RNA-binding proteins are core components of various ribonucleoprotein (RNP) complexes and are essential for posttranscriptional gene regulation (PTGR). RNA-binding proteins are involved in multiple aspects of RNA metabolism, including RNA splicing, RNA translocation, intracellular localization, and translation control [7]. Although RNA plays an indispensable role in RNA metabolism in organisms, its role in different diseases, especially in the development and progression of cancer, is still poorly understood. However, from the few relevant studies, it appears that RNA-binding proteins are closely related to both tumor origin and progression [7–9]. For instance, Zhang et al. found that a natural compound, neobractatin (*NBT*), significantly upregulates the expression of the RNA-binding protein Muscleblind-like 2 (*MBNL2*), which in turn inhibits tumor metastasis [10]. Another study on melanoma found that the RNA-binding protein *ELAVL1* was overexpressed in tumor tissue and significantly correlated with tumor progression and prognosis, promoting tumor formation and inhibiting cancer cell senescence [11]. The critical role of RBP has also been found in lung cancer research; for instance, Li et al. found that the expression of several essential RBP genes is strongly associated with the prognosis of lung adenocarcinoma [12]. However, the diversity of data from different sequencing platforms and the heterogeneity of tumors also affect the integration and analysis of large amounts of gene expression data. The standardization of crossplatform data is also a critical and challenging point for analysis. Recently, a new method based on relative sequencing of gene expression levels has been developed to overcome the shortcomings of traditional gene expression data processing and has yielded stable and reliable results in several studies [13–15].

In our research, the expression levels of a range of RBP-related genes within each tumor sample were compared in pairs using a novel method, ultimately generating a score for each gene pair [13, 15]. Scoring for this gene pair-based approach is based entirely on gene expression profiles within a single tumor sample. It does not need to be normalized across samples to account for differences between multiple samples or sequencing platforms [15]. We used The Cancer Genome Atlas (TCGA) RNA-seq dataset to construct the gene pair signature and to validate it by stratifying the dataset and by using the Gene Expression Omnibus (GEO) dataset. Subsequently, we confirmed the efficacy of this immunomarker in predicting tumor prognosis by comparing it with other clinicopathological information. The relationship between the signature and other prognosis-related factors, including tumor-infiltration lymphocyte cell content, tumor mutational burden (TMB), and tumor microenvironment, was further explored.

## 2. Materials and Methods

### 2.1. Data Sources of Lung Adenocarcinoma

The HTSeq-FPKM RNA-seq expression data, Masked Somatic Mutation data based on the “VarScan2 Variant Aggregation and Masking” workflow, and corresponding clinical data of 522 lung adenocarcinoma patient samples were retrieved from The Cancer Genome Atlas (TCGA) program dataset (https://portal. https://gdc.cancer.gov). Another validation dataset (GSE72094) was extracted from the Gene Expression Omnibus (GEO) database with corresponding survival information (http://www.ncbi.nlm. http://nih.gov/geo). The GSE72094 dataset of 442 lung adenocarcinoma patients was published on Oct 21, 2015, and based on the GPL15048 platform. Patients with overall survival time (OS) less than one month or missing survival information were excluded from the study. In total, 477 cases retrieved from TCGA database and 386 cases from the GEO database were recruited and analyzed in the present study.

### 2.2. Gene Expression Data Processing

The RNA-seq expression data was HTSeq-FPKM type. The expression profile data for each gene was converted to the corresponding gene symbol from the probe level according to the annotation file. No further standardization of the expressed data is required. If the patient has multiple samples, take the average expression value of each gene to represent the gene expression level of the patient. If there are multiple probes for a single gene, the average expression value will be taken as the expression level for that gene.

### 2.3. Modeling of the RBP-Related Gene Pair (IRGP) Signature

The extraction of RNA-binding protein-related genes was determined based on outstanding research published by Gerstberger et al.'s team in 2014. In total, they identified 1542 RNA-binding protein-related genes that are highly relevant to RNA metabolism [16]. We then calculated genes with differential expression between tumor samples and normal samples by applying the R package “limma.” We used these genes as candidate genes for the next gene pair analysis. The specific method of constructing the prognostic model is as described in the previous study [15]. Briefly, we performed a pairwise comparison to obtain a score for each gene pair between the gene expression values within each sample in the TCGA cohort. The score of a specific gene pair was set to one when the expression level of the first gene was higher than the other; otherwise was zero. We would discard the pairs if more than 90% of the scores of a pair were identity in the samples. We eventually constructed a signature of 33 IRGPs using the Lasso Cox proportional risk regression model. We then stratified patients into low- and high-risk groups using the most appropriate cutoff value. We used the “survivalROC” R package (using R package “survivalROC,” version 1.0.3) to obtain the cutoff value by the time-dependent receiver operating characteristic (ROC) curve analysis at three years for overall survival in TCGA dataset.

### 2.4. Prognostic Value of the Signature in the TCGA Cohort and Subcohorts

Survival analysis by the log-rank test was performed between the different immune risk groups. Subsequently, both univariate and multivariate Cox proportional hazard regression analyses of the risk factor and other clinical factors for the overall survival were performed in TCGA cohort. Pathologic stage and gender were converted as continuous variables. Stage I to stage IV were transformed into 1 to 4. We also randomly divided TCGA dataset into two different subcohorts and even split it into different subdatasets based on clinical characteristics such as age, gender, and pathological staging to verify the accuracy and validity of our model building in different subdatasets. Patients were divided into older and younger groups according to the median value of their age.

### 2.5. Dataset Validation of the Signature

To further prove the prognostic value of the signature in different cohorts, we applied the risk model to another independent cohort from the GEO database (GSE72094) for validation. We performed the same univariate and multivariate analyses as in TCGA cohort. Patients without matching clinical information will be excluded from the study.

### 2.6. Estimation of Immune Cell Abundance in Tumor Tissue

To analyze whether there were differences in the immune cell abundance of the tumor tissue in different risk groups, we used CIBERSORT (https://cibersort.stanford.edu/) to evaluate the relative fraction of predefined cell types in mixed solid tissues. The data used were normalized gene expression data of the tumor tissue [17]. We used the default LM22 leukocyte gene signature matrix from the CIBERSORT website. LM22 contains 547 genes distinguishing 22 types of immune-related cells. Disabling quantile normalization was checked. We set the number of permutations to 1000 for robust analyses. Then, CIBERSORT enumerated the relative proportions of the 22 infiltrating immune cells, including B cells, dendritic cells, T cells, natural killer cells, and macrophages.

### 2.7. Estimation of the Tumor Microenvironment (TME) and TMB

TMB usually refers to the number of somatic cell mutations detected per million bases, including gene coding errors, base substitutions, gene insertion, or deletion errors. After calculating TMB values for all samples, patients were grouped according to the previous risk cutoff values to explore whether there was a difference in TMB between high- and low-risk groups. TME is a general term for immune infiltrating cells and stromal cells in the tumor tissue other than tumor cells. Based on the RNA expression data, we used R package “estimate”(version 1.0.13) to score the immune microenvironment of all tumor tissue samples and scored the immune cell content and stromal cell content, respectively, to calculate the final tumor purity. We then grouped patients according to the previous prognostic model to compare whether there were differences in the TME between high- and low-risk groups.

### 2.8. Gene Ontology (GO) and the Kyoto Encyclopedia of Genes and Genomes (KEGG) Pathway Functional Enrichment Analysis and Gene Set Enrichment Analyses (GSEA)

GO and KEGG enrichment analysis was performed utilizing genes in the signature. We completed the GO and KEGG pathway analysis using R packages (“enrichplot,” “clusterProfiler,” and “ggplot2”) [18]. We used *P* value < 0.05 and *Q* − value < 0.05 as the threshold for GO and KEGG enrichment analysis. GSEA is used to assess the distribution trends of genes in a predefined set of genes in a gene set sequenced for phenotypic relevance and thus to determine their contribution to the phenotype [19]. We applied the GSEA software (Version 4.0.3, http://software.broadinstitute.org/gsea/) with 1,000 phenotype permutations for GSEA. The threshold of statistically significant gene sets was set to nominal *P* value < 0.05 with an FDR-adjusted *Q* − value < 0.25. We classified the patients into two groups according to their risk values. We then performed a GESA to compare whether there were pathways of differential enrichment between the two groups. MSigDB oncogenic signature gene sets (version 7.1, https://www.gsea-msigdb.org/gsea/downloads.jsp) were applied in the GSEA.

### 2.9. Protein-Protein Interaction (PPI) Network of Genes in the Signature

The genes that make up the prognostic model were used to construct the PPI network to analyze the intrinsic function of the model. The PPI network construction was based on the STRING database (https://string-db.org/), and we subsequently applied Cytoscape software (version 3.8.0) for the reconstruction and visualization of the network.

### 2.10. Statistical Analyses

Statistical analyses were mainly performed on R software (version 3.6.3, http://www.r-project.org). Survival analyses were performed using the “survival” package (version 3.1-11) with the Kaplan-Meier method. We used the Student two-sample *t*-test or Wilcoxon rank-sum test to compare the continuous variables. The “survival” package also calculated the RMS curve and time ratio. For all analyses, the statistical threshold was set to *P* value < 0.05.

## 3. Results

### 3.1. Differential Expression Analysis of RBP-Related Genes

**Figure 1:**
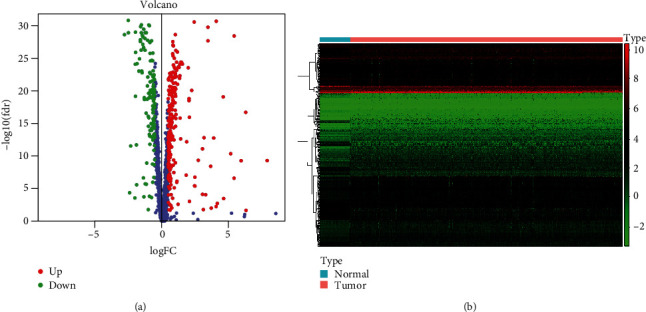
Abnormally expressed RBP genes existed in tumor tissues in TCGA. Heat map (a) and a volcano plot (b) of RBP gene expression.

A total of 1542 RBP-related genes that are highly relevant to RNA metabolism were recruited in the study. Overall, 379 RBP genes were differentially expressed in tumor tissues compared to normal tissues (FDR *P* value < 0.05 while LogFC value > 0.5). Two hundred forty-six genes were upregulated in expression, and 133 were downregulated in the tumor tissue (see Table S1 for details). The heat map and volcano plot of gene differential expression are detailed in Figures 1(a) and 1(b).

### 3.2. Construction of the RBP-Related Gene Pair Signature

**Figure 2:**
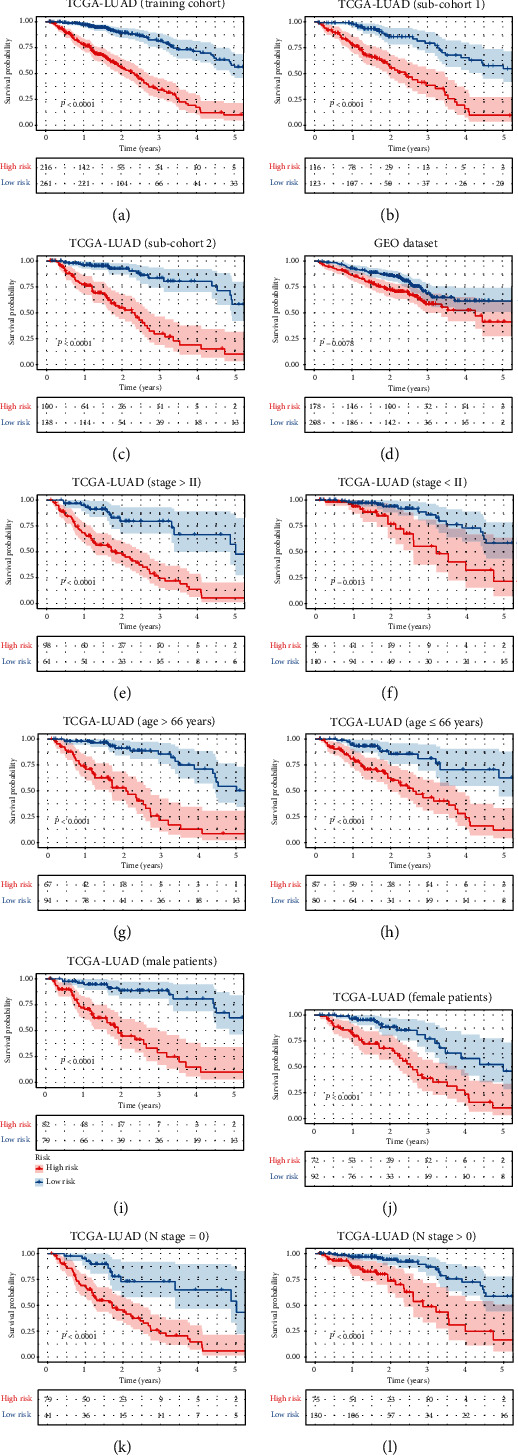
Survival curves for different risk groups in TCGA and GEO datasets. According to the optimal cutoff value, patients from different cohorts were stratified into the high- or low-risk group. Kaplan-Meier curves were used for survival analyses between different risk groups in different datasets: TCGA cohort (a), two TCGA validation subcohort (b, c), and the GEO validation cohort (d) groups stratified by pathologic stage (e, f), age (g, h), gender (i, j), and N stage (k, l).

**Figure 3:**
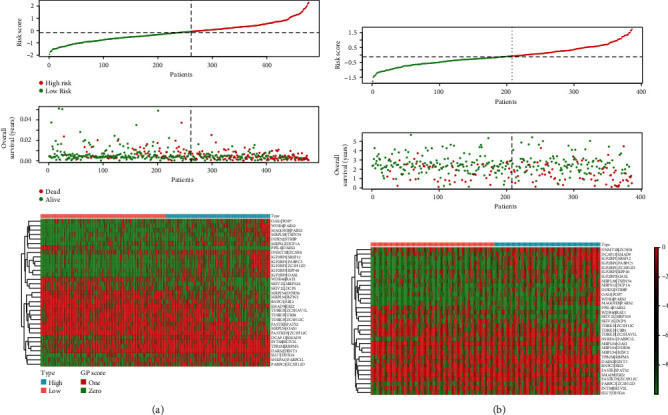
Plots of risk score analysis for the respective TCGA (a) (*n* = 477) and GEO (b) (*n* = 386) datasets. The plots in the first row were risk curves ranked by patient risk scores, and the plots in the middle row were survival status plots based on patient risk curves, which show a worse survival prognosis for the high-risk group. The heat maps at the bottom represent the patient's score for each gene pair. The score of a gene pair in a sample should be 0 or 1.

Gene expression data with corresponding clinical data of the TCGA cohort (*n* = 477) was used as an exploratory dataset. Genes with an average expression greater than 0 and genes with median absolute deviation (MAD) > 0.5 are included in the subsequent analysis. Patients with OS less than 30 days or without corresponding survival information were excluded. We used a total of 380 differentially expressed RBP genes as candidate genes for constructing the prognostic model. After rigorous screening to remove relatively small variation genes (MAD = 0), only 339 candidate genes were left for further study. Finally, a series of 33 gene pairs were recruited in the risk model using Lasso Cox proportional hazard regression from TCGA cohort (Table 1). Using the model, we can calculate a risk score for each sample. The fittest cutoff value of the IRGP risk score was set at −0.075 using a time-dependent ROC curve analysis. We then stratified the dataset into the high- or low-risk group according to the cutoff value (see Figure S1 in Supplementary Materials). Significantly, compared to the low-risk group, the high-risk group in the exploratory TCGA cohort exhibited an even worse OS (*P* < 0.0001, hazard ratio (HR) = 4.380 (3.139 to 6.111)) (Figure 2). The risk curves plotted in TCGA dataset based on model scores are shown in Figure 3(a). Significant differences in the pathologic tumor stage, T stage, and N stage were demonstrated between groups related to OS in the univariate Cox analysis (*P* < 0.001). However, in the multivariate Cox, only the phenotype of the signature exhibited a robust independent prognostic factor (*P* < 0.001) (see in Figure S2(a) and Table 2 for details). Highly similar results were found in the two randomly divided TCGA subcohort and other subcohorts stratified by age, gender, pathologic stage, and N stage. Risk subgroups obtained in each subcohort were consistently significantly correlated with survival prognosis (see in Figure 2).

### 3.3. Signature Validation in the GEO Dataset

Using the risk score cutoff, we stratified the patients in the GEO validation cohort into high- and low-risk groups. Consistent with the findings previously obtained in TCGA dataset, a significant difference of OS was found between the two groups (*P* = 7.8 × 10^−3^, HR = 1.597 (1.095 to 2.325)) (see in Figure 2). Notably, the RBP gene pair signature remained an independent predictive value of OS in both the univariate and multivariate Cox analyses in the validation dataset (*P* < 0.001) (see in Table 2 and Figure S2(b)). And the risk curves were similar to TCGA dataset, and high-risk patients also had a poorer prognosis (Figure 3(b)).

### 3.4. Immune Cell Infiltration between Different Risk Groups

**Figure 4:**
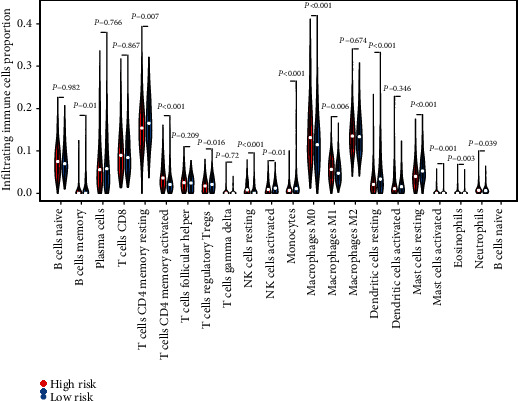
The relative fraction of infiltrated immune cells in different risk groups in TCGA dataset. Violin plot of differences in various immune cell abundances between the high- and low-risk groups. (^∗^*P* < 0.05, ^∗∗^*P* < 0.01, and ^∗∗∗^*P* < 0.001).

CIBERSORT was used to estimate the fractions of 22 infiltrated immune cells using the RNA-sequence data. We used a threshold of *P* < 0.05 to rule out unreliable results. Among the 535 tumor samples in TCGA, only 477 tumor samples were eligible for further analysis. The relative abundance of parts of the 22 infiltrated immune cells exhibited significant differences between the high- and low-risk groups (Figure 4). Compared to the low-risk group, the proportion of M0 macrophages, M1 macrophages, activated CD4 memory T cells, activated mast cells, eosinophils, and resting NK cells exhibited higher fraction in the high-risk group (*P* < 0.01). Conversely, memory B cells, resting dendritic cells, resting mast cells, monocytes, activated NK cells, regulatory T cells (Tregs), and restingCD4 memory T cells presented a significantly lower fraction in the high-risk group (*P* < 0.01) (Figure 4). Naive CD4 T cells were present in only two samples, so we did not include this type of cell in the statistical analysis.

### 3.5. Estimation of TME and TMB

**Figure 5:**
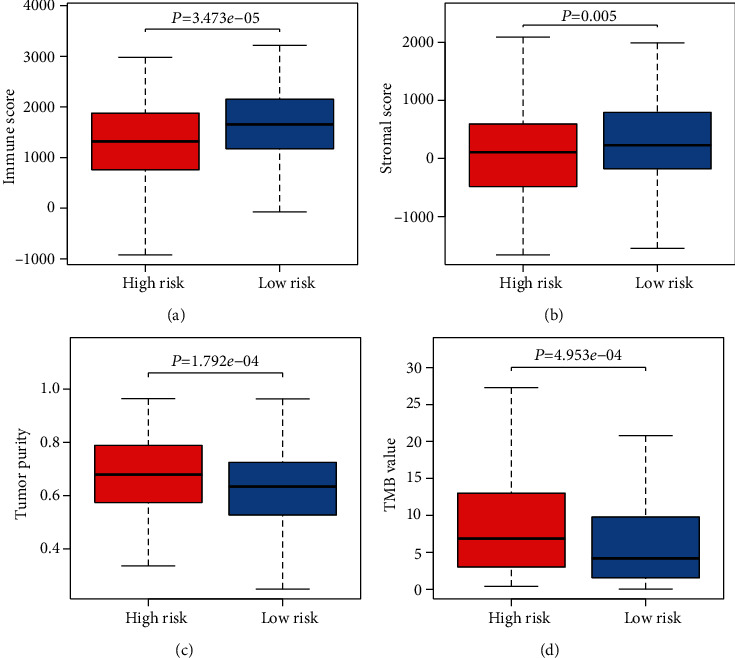
Analysis of differences in the tumor microenvironment (TME) and tumor mutational burden (TMB) between high- and low-risk groups: (a) immune score; (b) stromal score; (c) tumor purity; (d) tumor mutational burden.

Based on the prognostic risk model, we divided both TMB and TME data into high and low-risk groups and used the Wilcoxon signed-rank test to explore differences across risk groups. Notably, both the TMB- and TME-related indicators in the high and low-risk groups showed significant differences. Immune and stromal cells scored lower in tumor tissues in the high-risk group than in the low-risk group, and the corresponding average purity of tumors was higher than in the low-risk group (Figures 5(a)–5(c)). Besides, higher TMB values were present in the higher risk group than in the lower risk group (Figure 5(d)).

### 3.6. GO and KEGG Functional Analysis of Genes in the Signature

**Figure 6:**
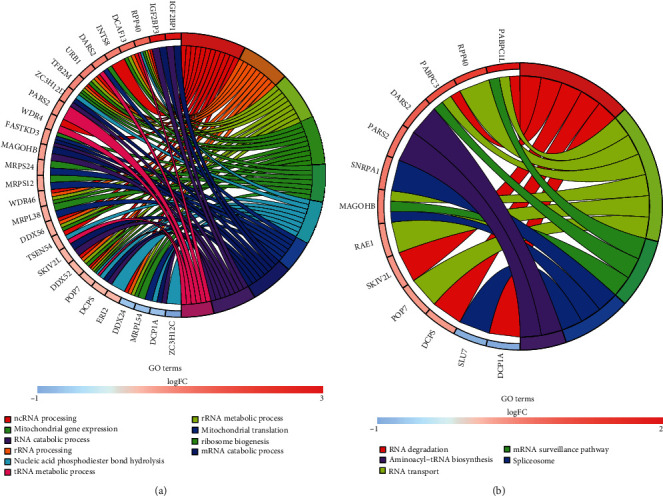
Circle plots of GO and KEGG enrichment analysis results for genes in the signature. (a) GO enrichment analysis showed that the function of these genes is mainly enriched in GO terms, including ncRNA processing and rRNA metabolic process. (b) KEGG enrichment analysis revealed that genes within the signature were predominantly enriched in five pathways, including RNA degradation (*P* < 0.05 and Q <0.05)). BP: biological process; CC: cellular component; MF: molecular function.

For the above 49 genes that make up the 33 genes pair prognostic model, we used GO and KEGG analyses to explore the closely related functions and pathways of these genes. Results of GO enrichment analysis revealed that the signature genes were enriched significantly in ncRNA processing, catalytic activity (acting on RNA), cytoplasmic ribonucleoprotein granule, and other GO terms (*P* < 0.05 and *Q* − value < 0.05) (Figure 6(a), Figure S3(a), and Figure S4). Results of the KEGG functional enrichment analysis revealed that genes in the signature were significantly enriched in five KEGG pathways (*P* < 0.05 and *Q* − value < 0.05). Among them, the pathways of RNA transport and RNA degradation were most significantly enriched (Figure 6(b), Figure S3(b), and Table S2).

### 3.7. GSEA Based on Risk Scoring

**Figure 7:**
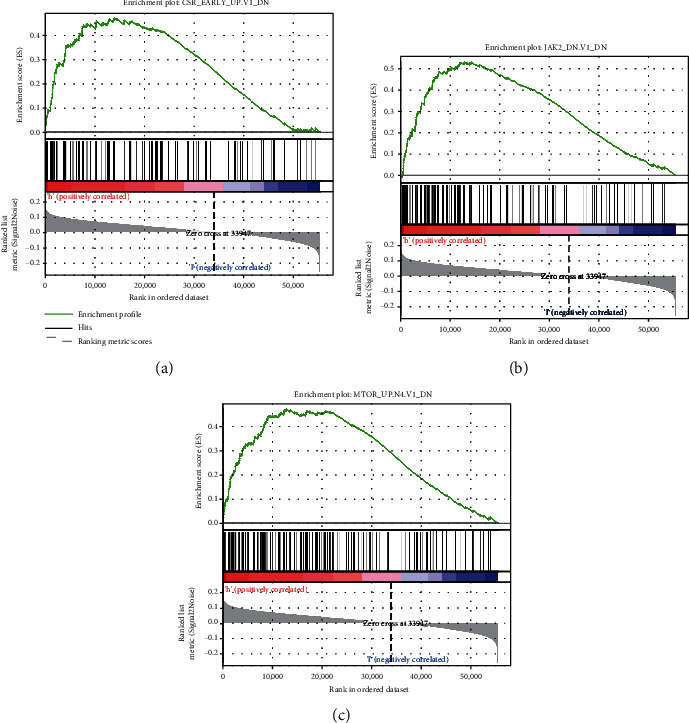
GSEA of TCGA cohort with oncogenic signature gene sets. According to the GSEA results, there were three significant gene set enrichments in the high-risk group (*P* < 0.05, FDR *Q* − value < 0.25). GSEA: gene set enrichment analysis.

Since the established signature was found to be highly correlated with prognosis, we then attempted to explore their functional implication and intrinsic association through enrichment analysis. We classified patients into high- and low-risk groups based on gene pair risk score cutoff value and used this risk classification as a phenotype for GSEA of TCGA cohort. As a result, we found the enrichment of three oncogenic signatures gene sets in the high-risk group, including “JAK2_DN.V1_DN,” “MTOR_UP.N4.V1_DN,” and “CSR_EARLY_UP.V1_DN” (significant at FDR *Q* − value < 25% and nominal *P* value < 1%) (Figure 7 and Table S3), which suggests a crucial role in lung adenocarcinoma progression and prognosis of these significantly enriched gene sets.

### 3.8. PPI Network Construction of the Genes in the Signature

**Figure 8:**
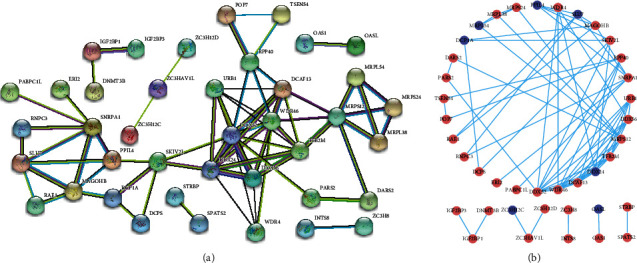
Protein-protein interaction (PPI) network analysis of the genes that make up the signature. (a) PPI network between the genes using STRING, with different edge colors representing various protein-protein associations. (b) The PPI network was plotted using Cytoscape, with red representing an upregulated expression of the gene in the tumor tissue and blue representing downregulation.

To better explore the potential function of the genes in the signature, we constructed a protein-protein interaction network using the STRING database. A total of 40 gene nodes and 69 edges are included in this PPI network (Figure 8 and Table S4). Most of the genes that make up the PPI network are overexpressed in the tumor tissue.

## 4. Discussion

As the most common pathological type of lung tumor, lung adenocarcinoma usually occurs around the lobe of the lung. In particular, a large proportion of lung cancers detected at an early stage are lung adenocarcinomas. The prognostic profile of lung adenocarcinoma is diverse, and overall, patients who are identified early and can undergo surgery survive much longer than patients who are no longer considered for surgery. Patients who can benefit from targeted therapy or immunotherapy have more prolonged survival and higher quality of life than those on regular chemotherapy, based on gene mutations and immunohistochemistry. According to previous studies, an excellent prognostic marker or model helps in predicting patient survival and better clinical management [20, 21]. Recently, Ling et al. used differentially expressed genes closely related to the tumor microenvironment to construct models to predict patient prognosis and explore the relationship between patient responsiveness to immunotherapy and the tumor microenvironment [21]. Zhao et al. created a tumor immunoscore clinical prognostic signature of lung adenocarcinoma using 109 immune-associated genes and validated its accuracy in different datasets [20]. However, the clinical applicability of these biomarkers remains limited due to tumor heterogeneity and sequencing technical problems. In particular, the issue of standardization of the data from different sequencing platforms is also a challenge in clinical applications. Therefore, in our study, to eliminate the influence of different platforms and interindividual standardization on the results, we introduced the concept of gene pairs. And by assigning the size of a particular pair of RBP gene expression values, we obtained a new predictive model that is more suitable for individual studies and clinical application. As described by Li et al., there is no need for data normalization or to consider technical bias across platforms as it only performs pairwise comparisons of the expression values of the target genes within a single sample [15]. Several previous studies have confirmed the availability and accuracy of this immune gene pair method in predicting overall survival in different types of cancer, including serous ovarian carcinoma and hepatocellular carcinoma [13–15, 22].

Although the exact mechanism is not yet clear, a growing body of research suggests that RBP plays an essential role in tumorigenesis and development [23, 24]. A study of glioblastoma found that expression levels of eight RBP-related genes were strongly associated with tumor prognosis, and identified PTRF and FNDC3B among them as potential prognosis-related biomarkers [23]. The RNA-binding protein PSF was found to play a pathophysiological role in ER-positive breast cancer by posttranscriptional regulation of the expression of its target genes ESR1 and SCFD2. Furthermore, PSF and SCFD2 were recognized as potential diagnostic and therapeutic targets for primary and hormone-refractory breast cancer [24]. To explore the function of RBPs in lung squamous cell carcinoma (LUSC), Li et al. obtained 300 RBP-associated genes that are aberrantly expressed in tumor tissues and ultimately screened 9 nine genes and successfully constructed a prognostic signature [7]. In our study, a robust signature of 33 RBP gene pairs that consists of 49 genes (Table 1) was identified to predict overall survival for lung adenocarcinoma patients. Some of these genes have been reported in previous studies to be associated with tumor development and prognosis [25, 26]. It was found that increased expression of INTS8 in the tumor tissue was associated with a more reduced overall survival and prognosis in patients with HCC. The authors suggested that the primary mechanism is to accelerate the epithelial-to-mesenchymal transition (EMT) by upregulating the TGF-*β* signaling pathway to promote tumor progression and metastasis [25]. IGF2BP1 also plays crucial roles in the generation and outcome of human tumors, such as the presence of high expression of IGF2BP1 in pancreatic tumor tissues, and its expression level is significantly associated with patient prognosis [27]. Similarly, DCAF13 has also been found to be highly expressed in a variety of tumors, including breast cancer, leading to a poorer tumor prognosis [28].

Tumor-infiltrating lymphocytes (TILs) are those leukocytes (NK cells, myeloid-derived suppressor cells (MDSCs), B cells, T cells, macrophages, dendritic cells (DCs), and others) that leave the bloodstream and enter the tumor tissue. Researchers have conducted relevant studies in many of these cancers, quantifying these tumor-infiltrating cells and correlating their abundance with tumor features and outcomes [29]. Previous studies have provided substantial evidence to support a favorable prognosis and outcome for malignant melanoma with abundant infiltration of TILs [30, 31]. In the current study, by using the CIBERSORT platform, we have estimated the relative fractions of 22 TILs in tumor tissues from TCGA cohort using the CIBERSORT platform. Using the prognostic signature cutoff, we divided the 22 TILs that resulted in high- and low-risk groups and compared whether differences exist in the content of each type of cell between the two groups. Significant differences in the relative fraction of infiltrated immune cells in tumor tissue were observed between the two different risk groups. In our study, we found more M1 macrophage, activated CD4 memory T cells, activated mast cells, and eosinophil infiltrates in the high-risk group.

Tumor-associated macrophages (TAM) not only prevent T cells from attacking tumor cells but also secrete growth factors that nourish tumor cells and promote tumor angiogenesis, leading to tumor cell expansion and metastasis [32–34]. M0 macrophage is an inactivated macrophage that, without any inflammatory or tumor-associated function, can be transformed into classically activated M1 macrophages and alternatively activated M2 macrophages. M1 macrophages have mainly antitumor effects and can differentiate tumor cells from healthy cells, recognize, and then kill tumor cells by mediating cytotoxic effects. The role of M2 macrophages is, on the contrary, to promote tumor growth and metastasis [32]. Both eosinophils and mast cells belong to inflammatory cells. These inflammatory cells are sought to contribute to barriers to antitumor immunity [35], because the inflammatory environment in the tumor tissue is believed to promote the development and progression of the tumor according to previous studies [36]. And conversely, we found that monocytes, activated NK cells, and Tregs presented a significantly lower fraction in the high-risk group. NK cells are critical immune cells in the body and are lymphocytes that can nonspecifically kill tumor cells and viral cells without presensitization. Lower levels in the tumor tissue predicted reduced tumor-killing capacity, favoring tumor progression, and a poorer prognosis.

Also, the high-risk group in our study had significantly increased TMB levels, which may suggest whether tumor patients could benefit from tumor immunotherapy, such as programmed death receptor 1 (PD-1) inhibitors, because, based on clinical and prior knowledge, patients with higher TMB values have a significantly higher response rate to immune checkpoint inhibitors than patients with lower TMB [37, 38]. According to the results of the tumor microenvironment in this study, tumor purity was significantly higher in the high-risk group than in the low-risk group. This suggests that the lower the amount of immune and stromal cells surrounding the tumor cells, the faster the tumor proliferates and progresses, and the worse the patient's prognosis may be. And this is relatively consistent with previous studies [20, 21].

Consistent with previous studies, GSEA of TCGA cohort revealed that three oncogenic signatures gene sets, including “MTOR_UP.N4.V1_DN,” were significantly enriched in the high-risk group, suggesting a crucial role in lung adenocarcinoma progression and metastasis of these gene sets. The present study found several JAK2-related genes in this signature. The JAK2-STAT signaling pathway is a cytokine-stimulated signal transduction pathway that has been identified in recent years and is involved in critical biological processes such as cell proliferation, apoptosis, and immune regulation [39]. The JAK2/STAT3 signaling pathway has been reported to be closely associated with the development and metastasis of non-small-cell lung cancer [40].

There are several limitations of the present study. The first is that the model built in this study is based on TCGA database. Although its feasibility was validated in a GEO dataset, it needs to be approved in more independent data in the future. Secondly, there is limited clinical data in public databases, and the impact of factors such as surgery, chemotherapy, and neoadjuvant therapy on clinical survival was not taken into account. These could have some effect on the accuracy of the results. Thirdly, there is a need to conduct functional analysis of genes in RBP-related prognostic models from multiple perspectives in the future to explore their intrinsic mechanisms related to survival, so as to provide more accurate clinical guidance.

## 5. Conclusions

Based on the differentially expressed RBP gene in the tumor tissue, we constructed a stable prognostic model for lung adenocarcinoma using a novel gene pair approach. We successfully validated its accuracy and availability in another independent dataset. This is strong evidence of the critical role of RBP in tumorigenesis and development. Notably, the method is based on pairwise comparison and assignment of different gene expressions within a single sample, which reduces the process and complexity of data processing for different platforms or sources and, therefore, can better help clinical treatment and management in the future.

## Figures and Tables

**Table 1:** Prognostic signature consists of 33 RBP gene pairs.

Gene A	Description	Gene B	Description	Coefficient
WDR46	WD repeat domain 46	RAE1	RAE1 RNA export 1 homolog (S. pombe)	-0.29314
SNRPA1	Small nuclear ribonucleoprotein polypeptide A′	PABPC1L	Poly(A) binding protein, cytoplasmic 1-like	0.248844
INTS8	Integrator complex subunit 8	SKIV2L	Superkiller viralicidic activity 2-like	0.039139
DCAF13	DDB1 and CUL4-associated factor 13	SMAD9	SMAD family member 9	0.048633
WDR4	WD repeat domain 4	PARS2	Prolyl-tRNA synthetase 2, mitochondrial (putative)	0.622802
MRPS12	Mitochondrial ribosomal protein S12	DCP1A	Decapping mRNA 1A	0.019737
IGF2BP1	Insulin-like growth factor 2 mRNA binding protein 1	PABPC3	Poly(A) binding protein, cytoplasmic 3	0.04353
IGF2BP1	Insulin-like growth factor 2 mRNA binding protein 1	SRSF12	Serine/arginine-rich splicing factor 12	0.214354
IGF2BP1	Insulin-like growth factor 2 mRNA binding protein 1	ZC3H12D	Zinc finger CCCH-type containing 12D	0.047965
IGF2BP3	Insulin-like growth factor 2 mRNA binding protein 3	RPP40	Ribonuclease P/MRP 40 kDa subunit	0.030426
IGF2BP3	Insulin-like growth factor 2 mRNA binding protein 3	OASL	2′-5′-oligoadenylate synthetase-like	0.098828
SLU7	SLU7 splicing factor homolog	DDX24	DEAD (Asp-Glu-Ala-Asp) box polypeptide 24	0.295423
MAGOHB	Mago-nashi homolog B (Drosophila)	PARS2	Prolyl-tRNA synthetase 2, mitochondrial (putative)	0.207791
MRPL38	Mitochondrial ribosomal protein L38	TSEN54	tRNA splicing endonuclease 54 homolog	0.1538
DDX52	DEAD (Asp-Glu-Ala-Asp) box polypeptide 52	STRBP	Spermatid perinuclear RNA-binding protein	0.012441
MRPL54	Mitochondrial ribosomal protein L54	OAS1	2′-5′-oligoadenylate synthetase 1, 40/46 kDa	-0.05311
MRPL54	Mitochondrial ribosomal protein L54	DDX56	DEAD (Asp-Glu-Ala-Asp) box helicase 56	-0.20468
MRPL54	Mitochondrial ribosomal protein L54	BZW2	Basic leucine zipper and W2 domains 2	-0.12583
SKIV2L	Superkiller viralicidic activity 2-like	MRPS24	Mitochondrial ribosomal protein S24	-0.22893
SKIV2L	Superkiller viralicidic activity 2-like	DCPS	Decapping enzyme, scavenger	-0.11877
OAS1	2′-5′-oligoadenylate synthetase 1, 40/46 kDa	POP7	Processing of precursor 7, ribonuclease P/MRP subunit	0.014735
RNPC3	RNA-binding region (RNP1, RRM) containing 3	ERI2	ERI1 exoribonuclease family member 2	-0.05978
DNMT3B	DNA (cytosine-5-)-methyltransferase 3 beta	ZC3H8	Zinc finger CCCH-type containing 8	0.139235
SMAD9	SMAD family member 9	ERI2	ERI1 exoribonuclease family member 2	-0.09502
TDRKH	Tudor and KH domain containing	URB1	URB1 ribosome biogenesis 1 homolog	-0.13488
TDRKH	Tudor and KH domain containing	ZC3HAV1L	Zinc finger CCCH-type, antiviral 1-like	-0.01879
TDRKH	Tudor and KH domain containing	ZC3H12C	Zinc finger CCCH-type containing 12C	-0.25482
PPIL4	Peptidylprolyl isomerase (cyclophilin)-like 4	DARS2	Aspartyl-tRNA synthetase 2, mitochondrial	-0.37219
TFB2M	Transcription factor B2, mitochondrial	RBPMS	RNA-binding protein with multiple splicing	0.029924
DARS2	Aspartyl-tRNA synthetase 2, mitochondrial	HINT3	Histidine triad nucleotide binding protein 3	0.126521
PABPC3	Poly(A) binding protein, cytoplasmic 3	ZC3H12D	Zinc finger CCCH-type containing 12D	0.157939
FASTK	Fas-activated serine/threonine kinase	SPATS2	Spermatogenesis associated, serine-rich 2-like	-0.04858
FASTKD3	FAST kinase domains 3	ZC3H12C	Zinc finger CCCH-type containing 12C	-0.06448

**Table 2:** Summary of the results of univariate and multivariate analyses of the risk factors for the overall survival of patients with lung adenocarcinoma in TCGA cohort and the GEO cohort.

Datasets	Variables	Univariate analysis		Multivariate analysis	
		HR (95% CI)	*P* value	HR (95% CI)	*P* value
TCGA (exploratory dataset)	Age	0.997 (0.978−1.015)	0.718	1.008 (0.989−1.028)	0.402
	Gender	1.000 (0.694−1.441)	1.000	0.980 (0.673−1.428)	0.917
	Stage	1.648 (1.396−1.946)	<0.001	2.119 (1.250-3.590)	0.005
	T stage	1.600 (1.285−1.994)	<0.001	1.083 (0.835-1.404)	0.549
	M stage	1.748 (0.959−3.187)	0.068	0.272 (0.071-1.039)	0.057
	N stage	1.787 (1.455−2.195)	<0.001	0.759 (0.472-1.233)	0.258
	Risk-score	3.967 (3.068−5.131)	<0.001	3.666 (2.791−4.815)	<0.001

GSE72094 (validation dataset)	Age	1.008 (0.985-1.031)	0.517	1.004 (0.980-1.028)	0.754
	Gender	1.885 (1.223-2.905)	0.004	2.205 (1.417-3.430)	<0.001
	Smoking	1.261 (0.549-2.897)	0.584	0.904 (0.390-2.095)	0.815
	Stage	1.704 (1.393-2.083)	<0.001	1.855 (1.499-2.297)	<0.001
	Risk-score	2.202 (1.525-3.179)	<0.001	2.214 (1.533-3.198)	<0.001

Abbreviations: HR: hazard ratio; CI: confidence interval.

## Data Availability

The data that support the findings of this study are openly available in TCGA at https://portal.gdc.cancer.gov/ and GEO database at http://www.ncbi.nlm.nih.gov/geo/.

## Supplementary Materials

Supplementary 1 Supplementary Figure 1: the optimal cutoff value of the risk score obtained by the time-dependent ROC curve analysis.

Supplementary 2 Supplementary Figure 2: forest plots of univariate and multivariate Cox regression analyses in different cohorts.

Supplementary 3 Supplementary Figure 3: functional clustering plots for GO and KEGG analyses.

Supplementary 4 Supplementary Figure 4: bar plot of the enriched GO terms in all categories for the RBP genes in the signature.

Supplementary 5 Supplementary Table 1: differential expression results of RBP-related genes between tumor and normal tissues.

Supplementary 6 Supplementary Table 2: results of KEGG enrichment analysis of genes in the signature.

Supplementary 7 Supplementary Table 3: risk score-based GSEA for MSigDB oncogenic signature gene sets.

Supplementary 8 Supplementary Table 4: PPI network analysis between genes in the signature.
