# Supplementary figures and images for: Predicting the Clinical Outcome of Lung Adenocarcinoma Using a Novel Gene Pair Signature Related to RNA-Binding Protein
Source: Biomed Res Int. 2020 Oct 26;2020:8896511. doi: 10.1155/2020/8896511 (PMC7643376; doi:10.1155/2020/8896511)

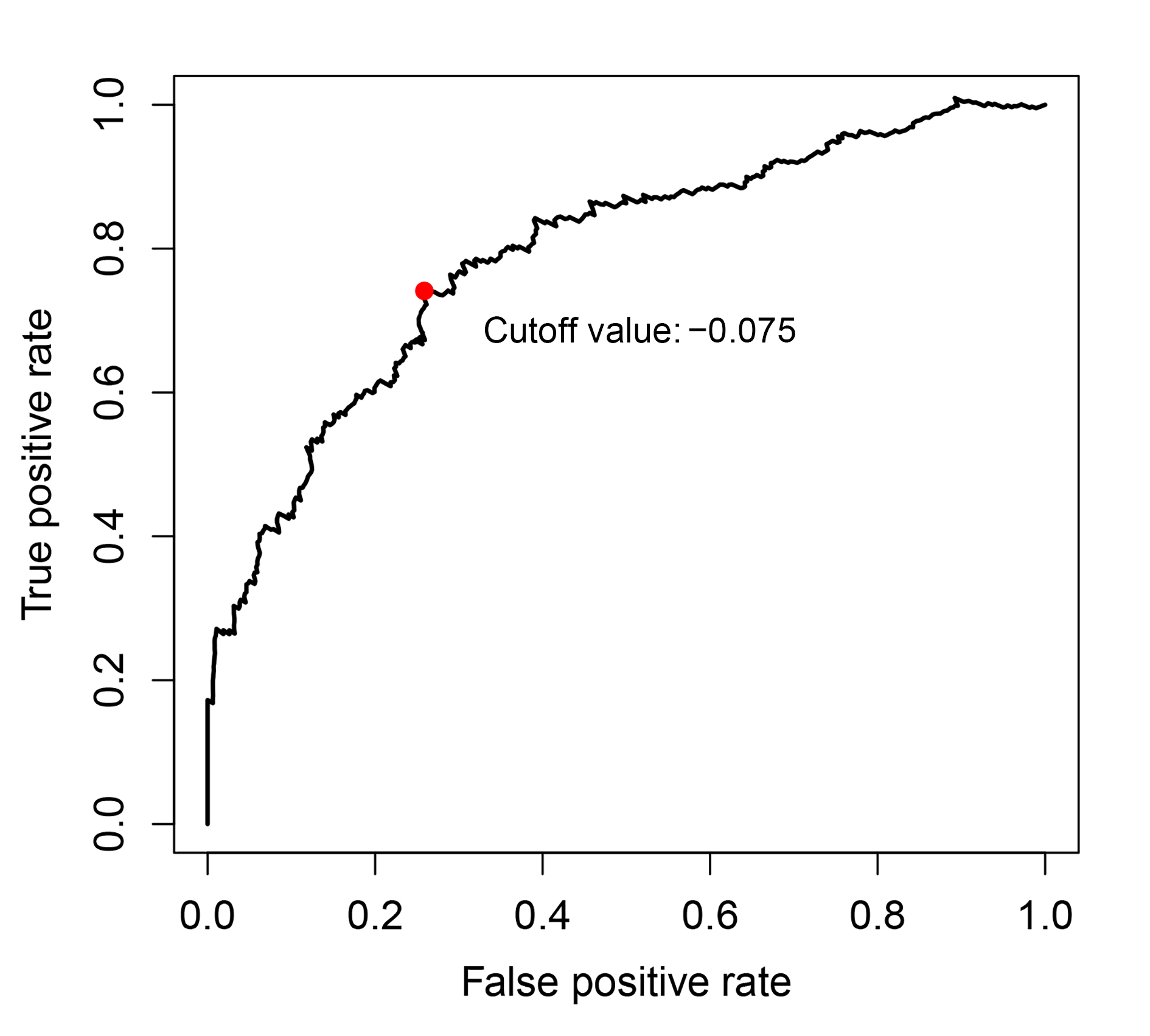

Supplement: Supplementary 1 — Supplementary Figure 1: the optimal cutoff value of the risk score obtained by the time-dependent ROC curve analysis. [file 8896511.f1.jpg]

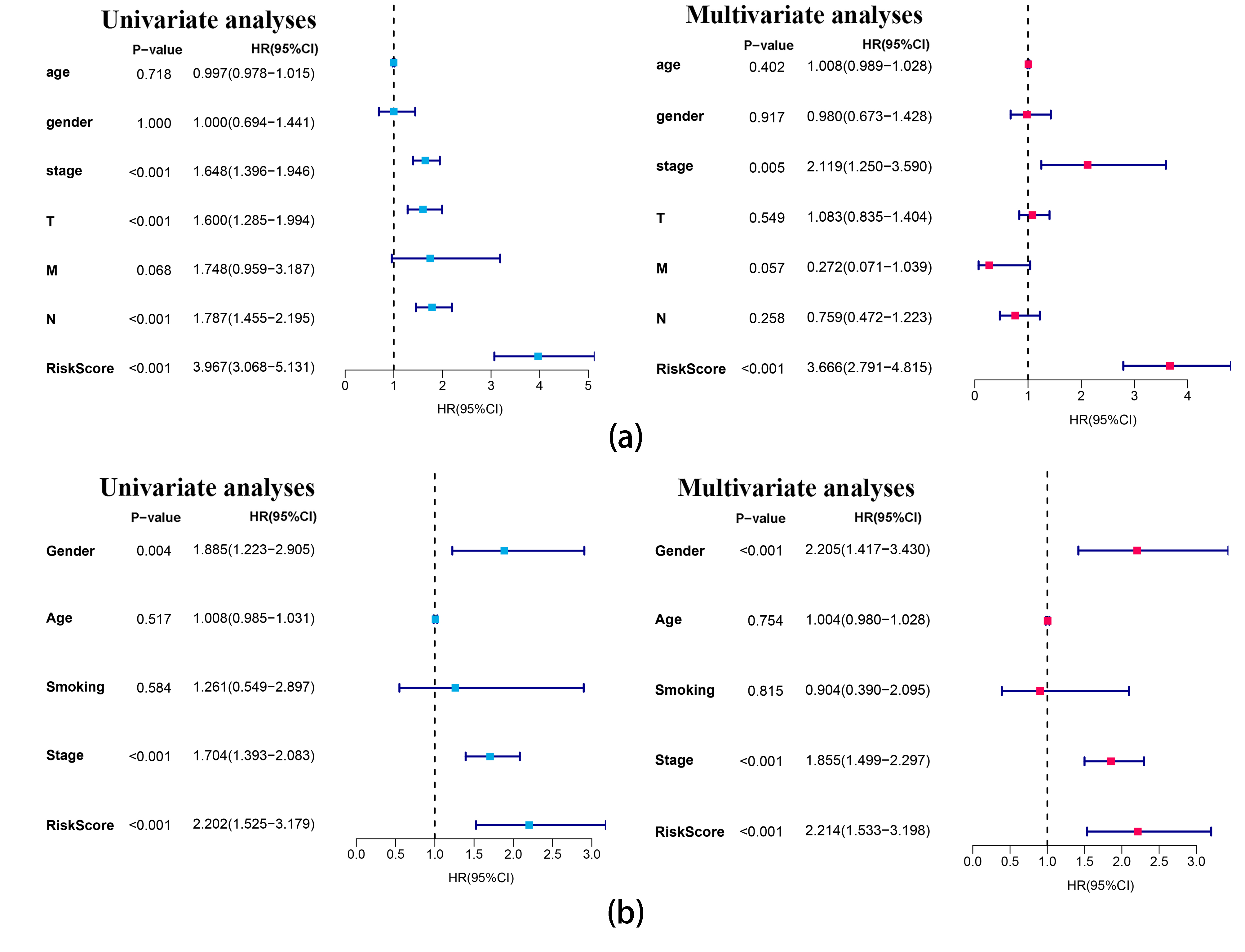

Supplement: Supplementary 2 — Supplementary Figure 2: forest plots of univariate and multivariate Cox regression analyses in different cohorts. [file 8896511.f2.jpg]

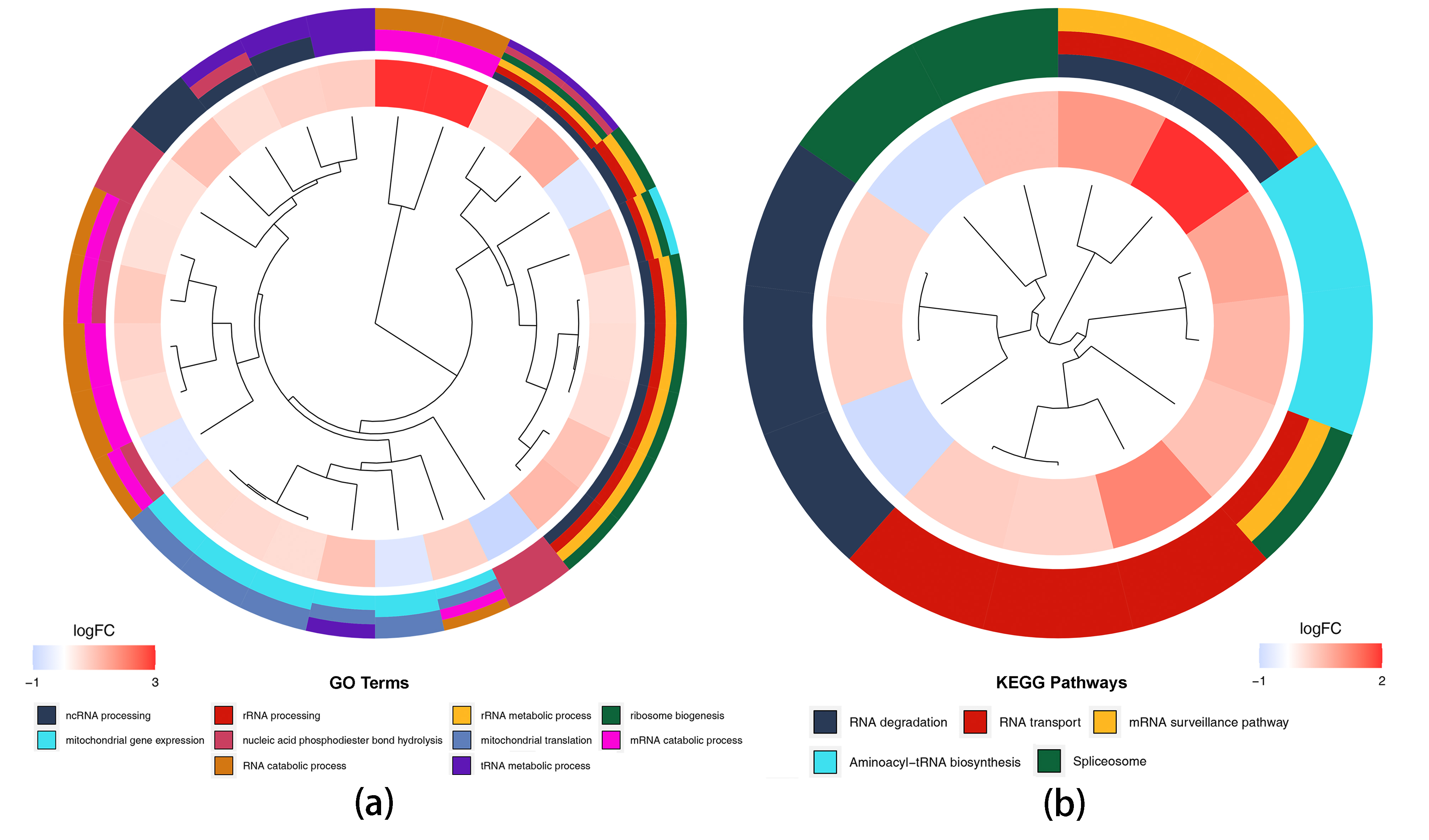

Supplement: Supplementary 3 — Supplementary Figure 3: functional clustering plots for GO and KEGG analyses. [file 8896511.f3.jpg]

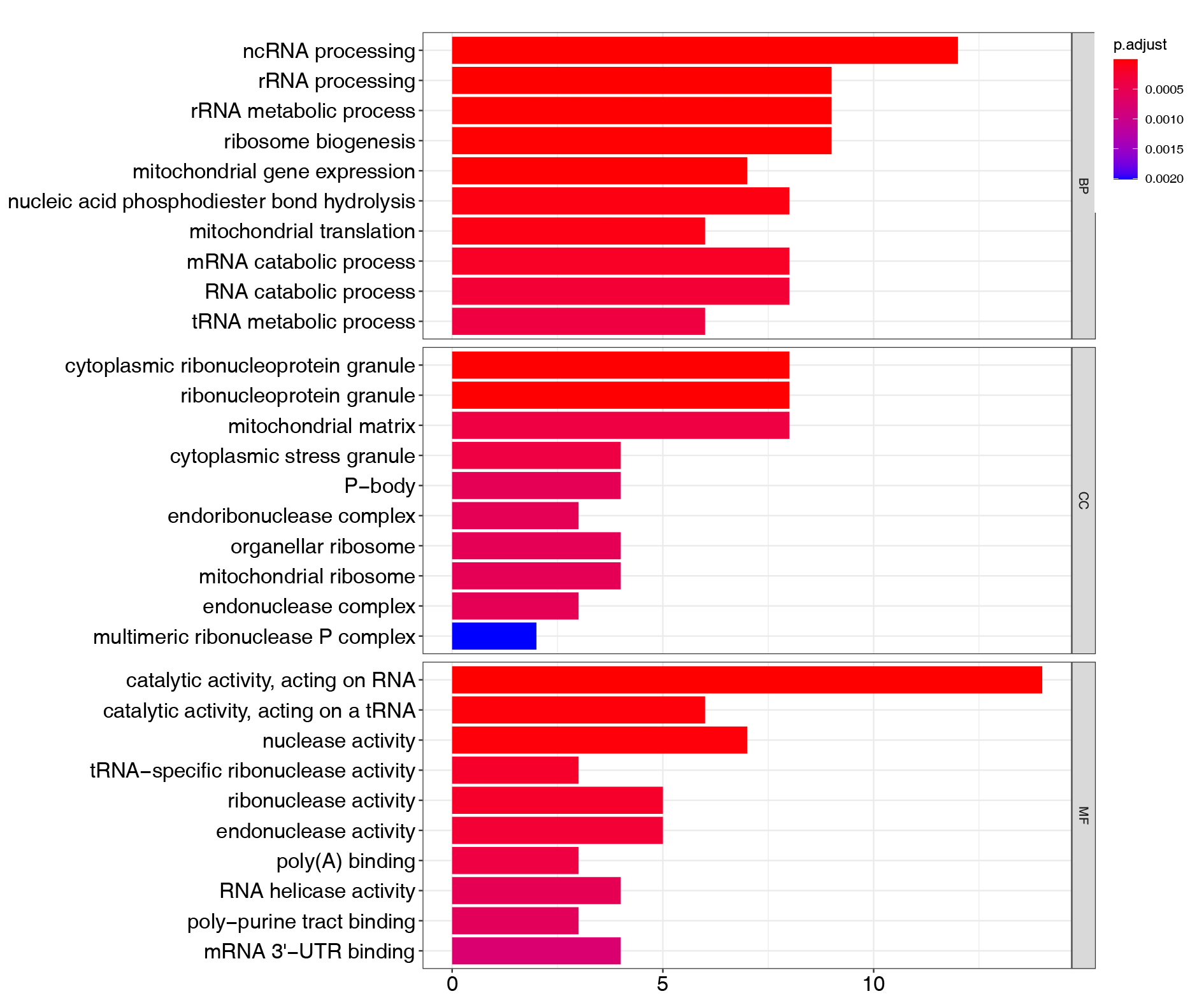

Supplement: Supplementary 4 — Supplementary Figure 4: bar plot of the enriched GO terms in all categories for the RBP genes in the signature. [file 8896511.f4.jpg]
