# Supplementary material for: Predicting the Clinical Outcome of Lung Adenocarcinoma Using a Novel Gene Pair Signature Related to RNA-Binding Protein
Source: Biomed Res Int. 2020 Oct 26;2020:8896511. doi: 10.1155/2020/8896511 (PMC7643376; doi:10.1155/2020/8896511)
Supplement: Supplementary 5 — Supplementary Table 1: differential expression results of RBP-related genes between tumor and normal tissues. [file 8896511.f5.docx]

Table S1 Differential expression results of RBP-related genes between tumor and normal tissues

| Genes | Mean (Normal) | Mean (Tumor) | LogFC | p-Value | FDR q-Value |
| --- | --- | --- | --- | --- | --- |
| *PSMA6* | 4.954776 | 7.131799 | 0.525446 | 2.93E-06 | 5.46E-06 |
| *TRIM56* | 14.25425 | 8.529952 | -0.74078 | 2.36E-15 | 1.17E-14 |
| *TRIM71* | 0.580561 | 0.178581 | -1.70087 | 4.58E-25 | 9.32E-24 |
| *WDR12* | 1.80372 | 3.294414 | 0.869047 | 6.62E-25 | 1.33E-23 |
| *MRPS34* | 39.56234 | 57.71781 | 0.544889 | 8.16E-14 | 3.28E-13 |
| *MSI2* | 1.927193 | 4.143514 | 1.104354 | 3.97E-24 | 6.39E-23 |
| *TRIT1* | 5.866626 | 9.166523 | 0.643844 | 3.37E-17 | 2.07E-16 |
| *MRPL36* | 8.37701 | 14.32038 | 0.773563 | 1.59E-14 | 7.17E-14 |
| *ZNFX1* | 17.40565 | 11.2188 | -0.63364 | 3.41E-17 | 2.08E-16 |
| *RBMS2* | 23.47618 | 8.257974 | -1.50734 | 2.21E-33 | 7.30E-31 |
| *RBMS1* | 13.10662 | 7.869123 | -0.73602 | 6.73E-27 | 2.07E-25 |
| *RBMS3* | 5.834585 | 1.945836 | -1.58424 | 2.27E-30 | 1.43E-28 |
| *CWF19L2* | 7.239733 | 4.650272 | -0.63862 | 2.04E-21 | 2.06E-20 |
| *L1TD1* | 0.646586 | 0.205767 | -1.65183 | 3.88E-27 | 1.22E-25 |
| *SRSF12* | 0.127631 | 0.541402 | 2.084717 | 1.78E-17 | 1.14E-16 |
| *DAP3* | 17.54234 | 26.1125 | 0.573899 | 1.16E-21 | 1.22E-20 |
| *TSFM* | 6.313085 | 10.05198 | 0.671062 | 2.41E-14 | 1.06E-13 |
| *STAU2* | 4.5659 | 6.919388 | 0.599745 | 1.45E-09 | 3.76E-09 |
| *WDR3* | 2.845711 | 4.248057 | 0.578014 | 7.69E-13 | 2.79E-12 |
| *CNOT11* | 16.55986 | 29.68876 | 0.842227 | 5.46E-30 | 2.78E-28 |
| *ELAVL2* | 0.021986 | 0.216673 | 3.300856 | 5.36E-05 | 9.07E-05 |
| *ELAVL4* | 0.037891 | 0.135917 | 1.842792 | 3.87E-08 | 8.77E-08 |
| *PUSL1* | 3.063729 | 6.208403 | 1.018934 | 2.88E-21 | 2.84E-20 |
| *ZGPAT* | 1.952845 | 2.95758 | 0.59884 | 2.51E-08 | 5.81E-08 |
| *TOP3B* | 0.06609 | 0.104833 | 0.665585 | 4.08E-07 | 8.29E-07 |
| *NCBP2L* | 0.160817 | 0.062689 | -1.35914 | 1.09E-07 | 2.34E-07 |
| *DDX56* | 13.7044 | 20.47936 | 0.579531 | 4.09E-21 | 3.97E-20 |
| *RBM28* | 1.505262 | 2.303493 | 0.613809 | 6.73E-19 | 4.97E-18 |
| *RPL37* | 60.76808 | 86.03328 | 0.501581 | 7.29E-10 | 1.95E-09 |
| *NOL10* | 6.442845 | 9.264228 | 0.523973 | 1.48E-23 | 2.08E-22 |
| *MRPL53* | 1.490559 | 2.295873 | 0.62319 | 7.53E-15 | 3.54E-14 |
| *ZC3H12B* | 0.456285 | 0.23294 | -0.96998 | 2.37E-21 | 2.37E-20 |
| *ZC3H12C* | 3.536939 | 1.729587 | -1.03207 | 5.00E-24 | 7.63E-23 |
| *ZC3H12D* | 0.401802 | 0.734969 | 0.871198 | 9.75E-10 | 2.57E-09 |
| *ZC3H12A* | 20.07586 | 10.07736 | -0.99434 | 0.013604 | 0.017363 |
| *EEF1G* | 0.124243 | 0.204288 | 0.717444 | 5.28E-05 | 8.95E-05 |
| *GARS* | 20.14232 | 33.76697 | 0.745383 | 1.44E-19 | 1.15E-18 |
| *TRMU* | 1.667457 | 2.981128 | 0.838209 | 3.20E-20 | 2.69E-19 |
| *CD3EAP* | 0.810801 | 1.683695 | 1.054212 | 7.03E-21 | 6.59E-20 |
| *RDM1* | 0.042366 | 0.470645 | 3.473649 | 3.64E-30 | 2.06E-28 |
| *BYSL* | 5.939838 | 11.74628 | 0.983709 | 8.50E-22 | 9.27E-21 |
| *XPO5* | 4.122249 | 7.446252 | 0.853083 | 1.21E-27 | 3.99E-26 |
| *DZIP1L* | 1.85017 | 1.15725 | -0.67696 | 6.73E-10 | 1.81E-09 |
| *GSPT2* | 6.190822 | 3.954339 | -0.64669 | 9.23E-14 | 3.67E-13 |
| *METTL1* | 4.310795 | 9.878902 | 1.196397 | 7.43E-23 | 9.53E-22 |
| *DARS2* | 5.040183 | 10.21585 | 1.019262 | 1.85E-25 | 4.01E-24 |
| *SAMD4A* | 4.868216 | 2.675809 | -0.86342 | 2.47E-15 | 1.22E-14 |
| *MRPL24* | 22.60779 | 37.25573 | 0.720642 | 1.58E-16 | 9.01E-16 |
| *IPO4* | 0.556984 | 1.380931 | 1.309934 | 1.12E-25 | 2.51E-24 |
| *ZNF598* | 6.348291 | 9.415099 | 0.568608 | 7.09E-13 | 2.58E-12 |
| *RCL1* | 3.929051 | 2.735725 | -0.52226 | 3.03E-16 | 1.67E-15 |
| *MRPL38* | 1.591746 | 2.401576 | 0.593371 | 8.10E-13 | 2.92E-12 |
| *BOP1* | 6.145422 | 16.7697 | 1.448273 | 1.36E-26 | 3.90E-25 |
| *MRPL12* | 12.19323 | 22.88741 | 0.908473 | 9.42E-16 | 4.80E-15 |
| *EIF6* | 56.21578 | 84.49629 | 0.587913 | 1.11E-14 | 5.14E-14 |
| *APOBEC1* | 0.004071 | 0.976072 | 7.905284 | 1.81E-10 | 5.20E-10 |
| *ZC3H7B* | 17.50798 | 11.38714 | -0.62061 | 1.33E-16 | 7.61E-16 |
| *WBP4* | 9.187255 | 5.471628 | -0.74766 | 3.73E-30 | 2.06E-28 |
| *BOLL* | 0.004701 | 0.039302 | 3.063485 | 4.57E-05 | 7.76E-05 |
| *DAZL* | 0.009093 | 0.050478 | 2.472786 | 1.99E-06 | 3.79E-06 |
| *SPATS2* | 2.488321 | 5.211891 | 1.066635 | 1.65E-28 | 6.40E-27 |
| *MAEL* | 0.095 | 1.250644 | 3.7186 | 0.007925 | 0.010324 |
| *RRP1* | 5.331838 | 8.130809 | 0.608766 | 6.58E-17 | 3.91E-16 |
| *TIPARP* | 31.93782 | 16.94494 | -0.91441 | 1.08E-14 | 5.02E-14 |
| *TDRD5* | 0.070512 | 0.900759 | 3.675209 | 1.50E-09 | 3.86E-09 |
| *POLR2J3* | 0.226532 | 0.427888 | 0.91752 | 1.51E-08 | 3.53E-08 |
| *BRIX1* | 4.445435 | 9.015076 | 1.020015 | 1.46E-24 | 2.56E-23 |
| *PWP2* | 0.157085 | 0.266781 | 0.764111 | 0.007408 | 0.009699 |
| *ZCCHC2* | 3.476317 | 2.411325 | -0.52773 | 1.10E-15 | 5.57E-15 |
| *THUMPD2* | 2.205333 | 3.29761 | 0.580424 | 2.31E-19 | 1.79E-18 |
| *MRPL34* | 23.0226 | 16.15879 | -0.51073 | 1.29E-10 | 3.77E-10 |
| *MRPS12* | 9.762419 | 15.26493 | 0.64491 | 5.20E-11 | 1.57E-10 |
| *EIF1* | 256.0426 | 153.6323 | -0.7369 | 1.33E-20 | 1.17E-19 |
| *EIF1B* | 30.04897 | 16.00973 | -0.90837 | 4.49E-33 | 9.90E-31 |
| *PLD6* | 1.688973 | 2.530691 | 0.583385 | 3.03E-07 | 6.22E-07 |
| *EXOSC4* | 9.528552 | 16.09801 | 0.756553 | 1.18E-12 | 4.13E-12 |
| *PTRH1* | 0.188987 | 0.103136 | -0.87374 | 4.35E-13 | 1.62E-12 |
| *INTS8* | 3.031627 | 6.18966 | 1.029768 | 2.63E-31 | 2.31E-29 |
| *MRPL54* | 38.70657 | 25.22478 | -0.61774 | 1.59E-16 | 9.01E-16 |
| *GTPBP3* | 2.332615 | 3.810796 | 0.708144 | 3.67E-16 | 1.99E-15 |
| *HINT3* | 25.42898 | 14.02906 | -0.85805 | 5.07E-28 | 1.76E-26 |
| *POLDIP3* | 36.56158 | 24.17572 | -0.59677 | 8.23E-23 | 1.05E-21 |
| *N4BP1* | 21.07837 | 9.035303 | -1.22212 | 1.26E-31 | 1.19E-29 |
| *NOP2* | 5.388802 | 9.875359 | 0.873868 | 3.99E-20 | 3.31E-19 |
| *MRPL15* | 25.46126 | 43.68443 | 0.778816 | 1.33E-16 | 7.61E-16 |
| *PUS1* | 2.052136 | 4.223928 | 1.041459 | 4.63E-23 | 6.11E-22 |
| *TDRKH* | 2.768017 | 6.543598 | 1.241231 | 9.67E-26 | 2.24E-24 |
| *OASL* | 10.29826 | 4.237253 | -1.2812 | 2.49E-20 | 2.11E-19 |
| *OAS1* | 10.21373 | 19.72618 | 0.949602 | 4.28E-06 | 7.86E-06 |
| *OAS3* | 10.69255 | 16.40277 | 0.617333 | 0.002085 | 0.00293 |
| *RNASE10* | 0.021208 | 0.119488 | 2.494165 | 8.26E-08 | 1.82E-07 |
| *CALR3* | 0.006994 | 0.039492 | 2.497443 | 2.21E-06 | 4.20E-06 |
| *PRPF3* | 7.474264 | 11.24093 | 0.588758 | 1.99E-11 | 6.18E-11 |
| *APOBEC4* | 1.677718 | 0.454855 | -1.88302 | 5.46E-13 | 2.01E-12 |
| *ZCCHC24* | 14.71316 | 6.240936 | -1.23727 | 1.43E-28 | 5.72E-27 |
| *DDX24* | 26.03344 | 17.21873 | -0.59639 | 3.49E-24 | 5.70E-23 |
| *MBNL2* | 26.12826 | 17.00173 | -0.61993 | 1.65E-22 | 2.04E-21 |
| *MBNL1* | 32.31958 | 22.2176 | -0.54071 | 1.38E-23 | 1.96E-22 |
| *PUS7* | 2.470351 | 5.391963 | 1.126095 | 1.40E-26 | 3.94E-25 |
| *PUS7L* | 1.111925 | 1.955498 | 0.814477 | 2.29E-19 | 1.79E-18 |
| *MRPS24* | 2.525019 | 3.980213 | 0.656551 | 4.18E-09 | 1.04E-08 |
| *NOL3* | 8.930757 | 14.15049 | 0.663998 | 2.27E-07 | 4.71E-07 |
| *EARS2* | 3.798933 | 5.90306 | 0.635869 | 4.28E-19 | 3.25E-18 |
| *SMAD9* | 5.167954 | 1.792383 | -1.52771 | 1.35E-28 | 5.57E-27 |
| *SMAD4* | 7.614871 | 5.131993 | -0.5693 | 1.95E-24 | 3.39E-23 |
| *SMAD7* | 21.89459 | 8.397327 | -1.38257 | 7.91E-31 | 5.50E-29 |
| *SMAD6* | 10.75412 | 1.568479 | -2.77745 | 2.82E-31 | 2.32E-29 |
| *ADARB2* | 0.238265 | 0.097383 | -1.29083 | 1.26E-20 | 1.12E-19 |
| *ADARB1* | 9.333315 | 2.410157 | -1.95326 | 7.34E-32 | 9.69E-30 |
| *CLK2* | 8.13355 | 14.18706 | 0.802619 | 1.07E-18 | 7.75E-18 |
| *DUS4L* | 0.951542 | 1.916833 | 1.010386 | 5.51E-24 | 8.27E-23 |
| *IGF2BP1* | 0.014823 | 0.919937 | 5.955591 | 1.90E-10 | 5.42E-10 |
| *IGF2BP3* | 0.115526 | 1.730241 | 3.904679 | 4.15E-14 | 1.76E-13 |
| *SNRPE* | 20.06076 | 34.50566 | 0.782457 | 4.70E-24 | 7.40E-23 |
| *NSUN5* | 5.585995 | 9.440054 | 0.756981 | 1.67E-19 | 1.31E-18 |
| *MTG1* | 0.935616 | 1.881863 | 1.008173 | 8.43E-20 | 6.87E-19 |
| *RPUSD1* | 5.510049 | 9.218453 | 0.742459 | 1.77E-16 | 1.00E-15 |
| *MRTO4* | 10.54437 | 16.19973 | 0.619497 | 8.54E-17 | 5.01E-16 |
| *MRPS33* | 4.685533 | 6.742277 | 0.525023 | 1.05E-10 | 3.10E-10 |
| *GAPDH* | 295.1779 | 751.81 | 1.348784 | 8.12E-24 | 1.19E-22 |
| *MOV10L1* | 0.056627 | 0.133561 | 1.237931 | 0.001873 | 0.002649 |
| *PARP1* | 18.06464 | 28.19331 | 0.642184 | 6.78E-18 | 4.43E-17 |
| *MRPS35* | 25.70725 | 37.0095 | 0.52572 | 1.59E-07 | 3.35E-07 |
| *AFF1* | 13.42302 | 9.323043 | -0.52584 | 2.46E-14 | 1.08E-13 |
| *AFF3* | 3.605716 | 0.645365 | -2.4821 | 1.18E-31 | 1.19E-29 |
| *MATR3* | 0.228858 | 0.469791 | 1.037566 | 4.10E-10 | 1.13E-09 |
| *SNRPD1* | 6.404816 | 9.311417 | 0.539844 | 3.46E-11 | 1.05E-10 |
| *FXR2* | 15.27144 | 9.668184 | -0.65952 | 2.94E-24 | 4.86E-23 |
| *CIRBP* | 57.15418 | 32.84942 | -0.79899 | 1.96E-21 | 1.99E-20 |
| *A1CF* | 0.00519 | 0.087954 | 4.082858 | 0.00444 | 0.005967 |
| *RBM46* | 0.006612 | 0.119267 | 4.172944 | 0.001356 | 0.00194 |
| *GPATCH4* | 5.530579 | 8.662138 | 0.647293 | 2.68E-15 | 1.31E-14 |
| *GNL3* | 14.77879 | 25.74861 | 0.800967 | 9.15E-25 | 1.73E-23 |
| *FAM120B* | 8.909722 | 5.760147 | -0.62927 | 3.94E-25 | 8.25E-24 |
| *KHDC1* | 0.43411 | 0.909774 | 1.067448 | 2.86E-17 | 1.77E-16 |
| *KHDC1L* | 0.013915 | 0.598664 | 5.427004 | 1.23E-07 | 2.61E-07 |
| *EPRS* | 22.32297 | 34.30289 | 0.619801 | 4.09E-16 | 2.20E-15 |
| *CCDC86* | 7.650539 | 11.66938 | 0.609095 | 5.96E-15 | 2.85E-14 |
| *C2orf15* | 1.03901 | 2.19383 | 1.078242 | 6.46E-18 | 4.24E-17 |
| *BARD1* | 1.199399 | 2.504345 | 1.062122 | 4.50E-22 | 5.26E-21 |
| *XPO4* | 5.099005 | 2.873023 | -0.82765 | 8.44E-27 | 2.48E-25 |
| *RUVBL1* | 5.68338 | 8.330238 | 0.551608 | 9.57E-13 | 3.41E-12 |
| *DDX52* | 3.078753 | 4.458969 | 0.534364 | 1.65E-18 | 1.16E-17 |
| *THOC1* | 2.104688 | 3.098445 | 0.557938 | 2.08E-11 | 6.43E-11 |
| *TRPT1* | 6.91244 | 11.17057 | 0.692435 | 7.28E-16 | 3.76E-15 |
| *NOP16* | 3.915321 | 6.367054 | 0.701495 | 7.42E-14 | 3.01E-13 |
| *DNMT3B* | 0.394606 | 1.576318 | 1.998074 | 1.30E-25 | 2.86E-24 |
| *ZC3HAV1L* | 1.157994 | 3.282671 | 1.503242 | 1.55E-26 | 4.27E-25 |
| *SIDT1* | 1.051409 | 1.866265 | 0.827831 | 0.000199 | 0.000312 |
| *SIDT2* | 17.41158 | 6.891265 | -1.33721 | 2.35E-32 | 3.45E-30 |
| *WDR4* | 2.481791 | 4.240724 | 0.772929 | 1.18E-19 | 9.47E-19 |
| *ATXN1L* | 10.58413 | 5.917366 | -0.83888 | 1.26E-24 | 2.28E-23 |
| *ILF2* | 60.13928 | 110.8934 | 0.882794 | 4.57E-26 | 1.10E-24 |
| *STRBP* | 3.048902 | 4.660211 | 0.612105 | 5.35E-16 | 2.83E-15 |
| *NXF5* | 0.022029 | 0.009454 | -1.22048 | 1.00E-06 | 1.96E-06 |
| *NXF2B* | 0.004508 | 0.001237 | -1.86581 | 1.41E-06 | 2.71E-06 |
| *NXF2* | 0.000389 | 0.000155 | -1.32278 | 0.000119 | 0.000191 |
| *NXF3* | 1.638751 | 0.420131 | -1.96368 | 3.18E-26 | 8.07E-25 |
| *RRS1* | 9.97124 | 16.00625 | 0.68279 | 2.07E-11 | 6.39E-11 |
| *PRKDC* | 8.510976 | 16.2028 | 0.928847 | 8.64E-14 | 3.45E-13 |
| *NOC2L* | 12.14213 | 17.52718 | 0.529572 | 3.23E-16 | 1.76E-15 |
| *NOL11* | 8.058104 | 11.42735 | 0.503978 | 1.88E-20 | 1.63E-19 |
| *PNPT1* | 5.541154 | 7.892619 | 0.510318 | 3.74E-13 | 1.41E-12 |
| *PIWIL1* | 0.002728 | 0.098661 | 5.176742 | 1.44E-11 | 4.54E-11 |
| *POP7* | 18.83631 | 27.10654 | 0.525125 | 4.50E-10 | 1.23E-09 |
| *FASTKD3* | 2.94235 | 4.97538 | 0.757838 | 9.13E-17 | 5.29E-16 |
| *TBRG4* | 6.273758 | 11.22133 | 0.838842 | 7.86E-24 | 1.17E-22 |
| *FASTK* | 14.16062 | 21.09754 | 0.575191 | 5.90E-11 | 1.77E-10 |
| *SARS2* | 1.131943 | 1.980025 | 0.806718 | 3.69E-14 | 1.58E-13 |
| *PAIP2* | 27.10551 | 17.29442 | -0.64828 | 4.45E-28 | 1.59E-26 |
| *PNRC2* | 41.21037 | 26.07157 | -0.66053 | 8.06E-27 | 2.42E-25 |
| *GEMIN6* | 2.359954 | 3.511221 | 0.573214 | 2.35E-12 | 8.01E-12 |
| *DDX39A* | 9.562664 | 15.13891 | 0.662777 | 6.85E-15 | 3.24E-14 |
| *DDX39B* | 10.78043 | 15.34302 | 0.509168 | 0.000968 | 0.001407 |
| *DHX37* | 2.973772 | 4.603931 | 0.630572 | 8.92E-17 | 5.21E-16 |
| *RNASE1* | 774.8841 | 387.9165 | -0.99823 | 1.27E-17 | 8.27E-17 |
| *RNASE2* | 1.349812 | 2.192497 | 0.699816 | 0.003021 | 0.00417 |
| *RNASE4* | 1.706749 | 0.99664 | -0.77611 | 1.86E-16 | 1.05E-15 |
| *ANG* | 13.54592 | 9.497165 | -0.51229 | 3.28E-14 | 1.42E-13 |
| *CTU2* | 2.955627 | 4.644611 | 0.652093 | 4.16E-15 | 2.03E-14 |
| *XPOT* | 9.547398 | 15.14343 | 0.665512 | 8.70E-13 | 3.10E-12 |
| *RBMXL1* | 6.777645 | 4.200331 | -0.69028 | 5.11E-22 | 5.92E-21 |
| *RBMXL2* | 0.027115 | 0.01446 | -0.90703 | 9.70E-05 | 0.000158 |
| *RBMY1J* | 0.004023 | 0.000767 | -2.39101 | 2.60E-05 | 4.49E-05 |
| *PPP1R10* | 25.58104 | 17.36712 | -0.55872 | 8.22E-10 | 2.18E-09 |
| *PRR3* | 2.674446 | 3.979511 | 0.573351 | 5.80E-16 | 3.05E-15 |
| *DKC1* | 10.12971 | 18.34694 | 0.856947 | 2.12E-29 | 9.64E-28 |
| *MRPL17* | 16.71209 | 23.65445 | 0.50122 | 3.04E-13 | 1.17E-12 |
| *DHX34* | 2.736791 | 4.850998 | 0.825796 | 2.52E-19 | 1.95E-18 |
| *HEXIM2* | 0.906636 | 1.294922 | 0.51427 | 2.26E-09 | 5.71E-09 |
| *SRRM3* | 0.147129 | 0.703199 | 2.25685 | 7.73E-22 | 8.59E-21 |
| *PIH1D3* | 1.912256 | 0.385399 | -2.31085 | 8.10E-13 | 2.92E-12 |
| *CSDC2* | 2.171109 | 1.458949 | -0.5735 | 8.99E-08 | 1.96E-07 |
| *RNPC3* | 1.784651 | 2.633251 | 0.561203 | 0.014768 | 0.018722 |
| *TRIM25* | 14.07577 | 7.814226 | -0.84904 | 7.87E-22 | 8.66E-21 |
| *MRPL9* | 18.93245 | 28.94096 | 0.612252 | 1.73E-22 | 2.12E-21 |
| *EXOSC5* | 7.35647 | 13.73488 | 0.900758 | 1.39E-17 | 8.94E-17 |
| *AIMP2* | 7.024787 | 12.22984 | 0.799879 | 7.54E-21 | 6.97E-20 |
| *BZW2* | 13.28044 | 27.113 | 1.029682 | 1.03E-24 | 1.91E-23 |
| *PARS2* | 1.722226 | 3.06141 | 0.829922 | 1.06E-20 | 9.45E-20 |
| *SKIV2L* | 8.504864 | 12.49334 | 0.554799 | 7.59E-17 | 4.47E-16 |
| *ZFP36* | 566.8754 | 146.6025 | -1.95112 | 7.36E-21 | 6.84E-20 |
| *ZFP36L2* | 87.87842 | 57.25713 | -0.61805 | 7.54E-16 | 3.88E-15 |
| *DROSHA* | 5.885582 | 9.191277 | 0.64308 | 3.42E-16 | 1.86E-15 |
| *PURA* | 4.790922 | 3.295325 | -0.53988 | 6.68E-22 | 7.54E-21 |
| *PURG* | 0.085429 | 0.046484 | -0.87801 | 2.08E-14 | 9.25E-14 |
| *TSEN54* | 8.053074 | 11.90537 | 0.564001 | 4.87E-15 | 2.36E-14 |
| *SRP9* | 72.99053 | 110.9743 | 0.604445 | 9.99E-13 | 3.54E-12 |
| *TARBP1* | 4.199616 | 7.043699 | 0.746076 | 1.63E-09 | 4.18E-09 |
| *AGO4* | 7.786849 | 5.296491 | -0.556 | 2.74E-16 | 1.52E-15 |
| *EZH2* | 0.886216 | 4.816402 | 2.442226 | 6.09E-34 | 2.68E-31 |
| *EIF3B* | 17.7381 | 27.38397 | 0.62648 | 1.33E-18 | 9.53E-18 |
| *HEATR1* | 3.033724 | 5.879181 | 0.954525 | 3.10E-27 | 1.00E-25 |
| *PTGES3L-AARSD1* | 0.016878 | 0.026285 | 0.639043 | 0.016557 | 0.02089 |
| *AARSD1* | 0.843462 | 1.565573 | 0.892296 | 2.64E-22 | 3.17E-21 |
| *CPSF3* | 8.830471 | 12.77049 | 0.532252 | 7.03E-22 | 7.87E-21 |
| *ETF1* | 50.64627 | 26.11393 | -0.95564 | 2.89E-33 | 7.64E-31 |
| *EIF2AK1* | 28.24148 | 46.66315 | 0.724468 | 8.84E-25 | 1.69E-23 |
| *KHDRBS2* | 3.80434 | 1.066981 | -1.83411 | 2.52E-26 | 6.67E-25 |
| *QKI* | 19.09769 | 6.456207 | -1.56464 | 7.31E-33 | 1.38E-30 |
| *PTCD2* | 0.611268 | 0.867282 | 0.504696 | 4.89E-11 | 1.48E-10 |
| *DDX55* | 2.541841 | 4.175336 | 0.716019 | 2.05E-22 | 2.49E-21 |
| *CPSF4* | 4.857831 | 7.238101 | 0.575299 | 2.82E-13 | 1.09E-12 |
| *CSTF2* | 5.646683 | 10.41035 | 0.882543 | 2.79E-29 | 1.23E-27 |
| *RAVER2* | 5.183644 | 2.857251 | -0.85934 | 7.77E-21 | 7.13E-20 |
| *NSUN2* | 11.54455 | 19.5897 | 0.762883 | 2.63E-21 | 2.61E-20 |
| *URB2* | 2.137078 | 3.201158 | 0.582954 | 5.80E-14 | 2.40E-13 |
| *PPARGC1A* | 1.567137 | 0.935934 | -0.74365 | 2.78E-14 | 1.21E-13 |
| *PPARGC1B* | 1.289417 | 0.469067 | -1.45885 | 8.77E-25 | 1.69E-23 |
| *MCTS1* | 8.170872 | 12.86023 | 0.654354 | 8.24E-19 | 6.02E-18 |
| *ZC3H13* | 10.31063 | 6.528911 | -0.65922 | 2.74E-19 | 2.11E-18 |
| *RBM24* | 0.561444 | 0.25212 | -1.15503 | 3.99E-18 | 2.69E-17 |
| *THOC3* | 1.479594 | 2.615729 | 0.822011 | 2.06E-14 | 9.18E-14 |
| *VARS* | 11.98925 | 22.17365 | 0.887105 | 5.32E-23 | 6.96E-22 |
| *INTS7* | 4.032962 | 6.625421 | 0.716172 | 3.79E-21 | 3.71E-20 |
| *PPIL4* | 14.7202 | 10.34416 | -0.50898 | 1.38E-19 | 1.11E-18 |
| *CTIF* | 9.855943 | 3.653422 | -1.43175 | 4.31E-31 | 3.35E-29 |
| *C9orf129* | 0.051526 | 0.032611 | -0.65995 | 5.43E-07 | 1.09E-06 |
| *RBPMS* | 16.58135 | 10.41545 | -0.67084 | 2.92E-16 | 1.62E-15 |
| *RPL3L* | 0.03252 | 0.119662 | 1.879566 | 1.68E-13 | 6.60E-13 |
| *RNASE11* | 0.000258 | 6.46E-05 | -1.99734 | 0.000178 | 0.000281 |
| *RBM34* | 0.545932 | 0.774658 | 0.504839 | 5.62E-08 | 1.25E-07 |
| *ENDOU* | 0.257839 | 0.092024 | -1.48638 | 4.43E-25 | 9.15E-24 |
| *TYW5* | 0.800227 | 1.181266 | 0.561853 | 1.32E-11 | 4.16E-11 |
| *RAE1* | 4.078365 | 6.06697 | 0.572985 | 2.07E-17 | 1.31E-16 |
| *SLU7* | 15.4504 | 10.30777 | -0.58391 | 2.25E-24 | 3.86E-23 |
| *EEF1A1* | 1058.826 | 743.6191 | -0.50983 | 1.13E-19 | 9.18E-19 |
| *EEF1A2* | 0.441219 | 34.79853 | 6.301387 | 2.92E-18 | 2.01E-17 |
| *R3HDM1* | 2.65708 | 4.321607 | 0.701726 | 8.56E-22 | 9.27E-21 |
| *ERI2* | 1.673418 | 2.383838 | 0.510488 | 4.82E-12 | 1.60E-11 |
| *NIFK* | 11.65091 | 16.55022 | 0.506408 | 4.70E-16 | 2.52E-15 |
| *WDR46* | 11.92886 | 18.06988 | 0.599131 | 5.02E-24 | 7.63E-23 |
| *CMSS1* | 3.149399 | 4.475981 | 0.507127 | 1.23E-07 | 2.61E-07 |
| *ZNF106* | 17.77825 | 6.215015 | -1.51628 | 1.10E-31 | 1.19E-29 |
| *UTP14A* | 4.41866 | 6.760889 | 0.613604 | 1.13E-24 | 2.08E-23 |
| *UTP14C* | 7.753318 | 5.240499 | -0.56511 | 2.17E-20 | 1.86E-19 |
| *TRUB2* | 4.428881 | 6.608396 | 0.577358 | 8.74E-15 | 4.08E-14 |
| *PRIM1* | 2.349458 | 3.830787 | 0.705313 | 1.44E-06 | 2.75E-06 |
| *RNASE13* | 0.035928 | 0.016655 | -1.10912 | 4.55E-10 | 1.24E-09 |
| *MRPL3* | 21.10154 | 34.71788 | 0.718331 | 1.40E-24 | 2.49E-23 |
| *TFB2M* | 6.913256 | 13.35813 | 0.950281 | 1.06E-25 | 2.41E-24 |
| *CPSF1* | 12.05601 | 18.87466 | 0.646698 | 1.90E-09 | 4.85E-09 |
| *DDX3X* | 56.88356 | 36.59378 | -0.63641 | 3.51E-18 | 2.39E-17 |
| *DDX4* | 0.005479 | 0.036855 | 2.749862 | 1.61E-10 | 4.66E-10 |
| *NOP56* | 12.05836 | 17.39698 | 0.528804 | 6.79E-13 | 2.48E-12 |
| *MRM1* | 2.269247 | 4.986478 | 1.135807 | 3.08E-25 | 6.55E-24 |
| *RPL21* | 122.1525 | 78.64354 | -0.63528 | 4.55E-24 | 7.24E-23 |
| *NOL12* | 1.124721 | 1.71589 | 0.609389 | 6.48E-09 | 1.56E-08 |
| *SNRPA1* | 4.973237 | 8.43577 | 0.762335 | 2.20E-23 | 3.02E-22 |
| *FTO* | 6.658707 | 3.756971 | -0.82567 | 9.04E-22 | 9.63E-21 |
| *SAMHD1* | 72.36594 | 28.47385 | -1.34567 | 5.23E-29 | 2.23E-27 |
| *PCF11* | 7.219426 | 5.032107 | -0.52072 | 6.93E-13 | 2.53E-12 |
| *NPM3* | 9.325771 | 24.10225 | 1.369873 | 3.33E-26 | 8.29E-25 |
| *NUPL2* | 4.112886 | 6.450562 | 0.649274 | 5.82E-18 | 3.86E-17 |
| *SF3A1* | 31.31652 | 21.23973 | -0.56016 | 5.17E-22 | 5.93E-21 |
| *TDRD3* | 4.660988 | 2.578218 | -0.85426 | 3.09E-23 | 4.16E-22 |
| *SNRPB* | 89.43181 | 137.4029 | 0.619553 | 2.41E-11 | 7.43E-11 |
| *SNRPN* | 25.9542 | 17.40962 | -0.57608 | 1.20E-14 | 5.49E-14 |
| *ERN2* | 0.665914 | 5.814911 | 3.126349 | 0.013648 | 0.017403 |
| *GTPBP4* | 6.607368 | 9.67804 | 0.550639 | 1.53E-12 | 5.31E-12 |
| *MRPL13* | 5.699574 | 8.840178 | 0.633221 | 8.42E-13 | 3.02E-12 |
| *LSM5* | 5.405599 | 7.80057 | 0.529125 | 6.50E-12 | 2.12E-11 |
| *PIH1D2* | 1.693869 | 1.009169 | -0.74715 | 0.000151 | 0.00024 |
| *TTF2* | 1.240403 | 2.155955 | 0.797518 | 4.16E-22 | 4.91E-21 |
| *UTP3* | 22.62306 | 15.53533 | -0.54224 | 1.11E-23 | 1.60E-22 |
| *PTBP2* | 0.882576 | 1.345682 | 0.608544 | 6.48E-06 | 1.16E-05 |
| *TLR7* | 2.849042 | 1.473561 | -0.95117 | 2.18E-17 | 1.38E-16 |
| *TLR8* | 7.386716 | 1.918146 | -1.94522 | 3.35E-28 | 1.23E-26 |
| *TLR3* | 4.582603 | 2.729134 | -0.74772 | 1.22E-15 | 6.14E-15 |
| *TRMT2A* | 5.969712 | 8.925963 | 0.580347 | 6.61E-12 | 2.15E-11 |
| *BRCA1* | 0.770555 | 2.015883 | 1.387442 | 2.47E-17 | 1.53E-16 |
| *DQX1* | 0.06021 | 0.503943 | 3.065188 | 2.40E-12 | 8.14E-12 |
| *SAP18* | 42.0516 | 29.04698 | -0.53377 | 5.60E-22 | 6.38E-21 |
| *TDRD12* | 0.051299 | 0.218119 | 2.08811 | 0.000647 | 0.000955 |
| *URB1* | 2.732337 | 5.519345 | 1.014361 | 2.70E-22 | 3.22E-21 |
| *CELF2* | 22.72605 | 8.609386 | -1.40036 | 1.55E-29 | 7.31E-28 |
| *CELF3* | 0.031847 | 0.797617 | 4.646455 | 0.000223 | 0.000348 |
| *CELF5* | 0.028636 | 0.258799 | 3.175953 | 3.68E-14 | 1.58E-13 |
| *CPEB1* | 0.30015 | 0.144121 | -1.0584 | 1.39E-22 | 1.75E-21 |
| *CPEB2* | 5.64418 | 3.76609 | -0.5837 | 5.33E-17 | 3.22E-16 |
| *CPEB3* | 1.496081 | 0.982474 | -0.6067 | 4.39E-20 | 3.63E-19 |
| *CPEB4* | 8.154376 | 5.406304 | -0.59293 | 6.30E-17 | 3.76E-16 |
| *PRPF8* | 40.51449 | 28.26059 | -0.51965 | 8.73E-15 | 4.08E-14 |
| *SECISBP2L* | 30.14473 | 10.0645 | -1.58263 | 5.06E-30 | 2.67E-28 |
| *EEF1E1* | 3.500736 | 5.299393 | 0.598169 | 7.90E-12 | 2.55E-11 |
| *TARS* | 11.63237 | 18.9503 | 0.704077 | 8.61E-14 | 3.45E-13 |
| *TARS2* | 5.823864 | 10.36329 | 0.831433 | 5.86E-28 | 1.99E-26 |
| *IARS* | 12.36218 | 17.5142 | 0.502592 | 1.80E-13 | 7.04E-13 |
| *APEX1* | 56.50071 | 79.91329 | 0.500166 | 3.46E-14 | 1.49E-13 |
| *PABPC3* | 0.151678 | 0.337377 | 1.153355 | 3.74E-07 | 7.63E-07 |
| *PABPC1L* | 1.832719 | 7.622645 | 2.056306 | 3.84E-20 | 3.21E-19 |
| *PABPC5* | 0.478497 | 0.242339 | -0.98148 | 5.25E-16 | 2.80E-15 |
| *PABPC1* | 206.2705 | 363.3337 | 0.816758 | 5.88E-14 | 2.42E-13 |
| *MEX3A* | 0.294937 | 5.039136 | 4.094697 | 3.08E-34 | 2.03E-31 |
| *MEX3B* | 0.571688 | 1.182976 | 1.049122 | 5.28E-16 | 2.80E-15 |
| *MEX3D* | 6.367077 | 9.847477 | 0.629123 | 5.48E-08 | 1.22E-07 |
| *GEMIN7* | 5.695643 | 8.606829 | 0.595623 | 4.77E-13 | 1.76E-12 |
| *MRPS23* | 6.790059 | 9.693643 | 0.513615 | 1.65E-09 | 4.22E-09 |
| *PSIP1* | 13.33 | 8.141633 | -0.71129 | 9.50E-21 | 8.54E-20 |
| *FARSB* | 9.427112 | 13.66831 | 0.535947 | 1.80E-18 | 1.26E-17 |
| *RPL39L* | 3.461552 | 14.42196 | 2.058776 | 1.81E-20 | 1.57E-19 |
| *SNRNP25* | 6.088842 | 9.752167 | 0.679555 | 5.80E-14 | 2.40E-13 |
| *GEMIN2* | 2.563641 | 4.139748 | 0.691349 | 1.02E-09 | 2.67E-09 |
| *UTP20* | 2.677582 | 4.333353 | 0.694553 | 1.37E-07 | 2.89E-07 |
| *SRSF5* | 49.10647 | 31.5713 | -0.6373 | 3.15E-16 | 1.73E-15 |
| *TERT* | 0.005656 | 0.244743 | 5.435395 | 5.05E-31 | 3.70E-29 |
| *ZC3H8* | 0.952122 | 1.620389 | 0.767122 | 1.19E-21 | 1.25E-20 |
| *DCAF13* | 2.797576 | 6.359082 | 1.184641 | 2.81E-28 | 1.06E-26 |
| *ARHGEF28* | 2.14253 | 1.438466 | -0.57478 | 4.15E-12 | 1.38E-11 |
| *PRPF40B* | 0.648901 | 1.154797 | 0.831568 | 2.28E-17 | 1.42E-16 |
| *EIF3L* | 48.67818 | 33.20144 | -0.55203 | 5.45E-19 | 4.04E-18 |
| *POP1* | 1.198113 | 1.969013 | 0.716708 | 2.58E-16 | 1.44E-15 |
| *DCPS* | 4.189381 | 6.009016 | 0.520392 | 1.93E-14 | 8.64E-14 |
| *POLR2H* | 8.974864 | 16.72382 | 0.897943 | 4.43E-26 | 1.08E-24 |
| *ZNF239* | 0.883298 | 2.604922 | 1.560268 | 3.03E-26 | 7.84E-25 |
| *YBX2* | 0.053997 | 1.324729 | 4.616675 | 9.41E-21 | 8.51E-20 |
| *YBX3* | 29.84297 | 19.90132 | -0.58453 | 9.07E-17 | 5.28E-16 |
| *NSRP1* | 9.777121 | 6.853971 | -0.51247 | 4.58E-19 | 3.46E-18 |
| *TRIM21* | 24.17618 | 14.92781 | -0.69558 | 2.19E-23 | 3.02E-22 |
| *EED* | 2.150718 | 3.133974 | 0.543175 | 5.94E-16 | 3.10E-15 |
| *SBDS* | 96.84993 | 47.22379 | -1.03624 | 2.92E-30 | 1.75E-28 |
| *RPL22L1* | 6.73402 | 12.59711 | 0.903552 | 1.47E-10 | 4.25E-10 |
| *RNASEH2A* | 5.296753 | 10.76925 | 1.023738 | 1.36E-18 | 9.66E-18 |
| *FRG1* | 16.53255 | 11.37031 | -0.54004 | 1.72E-21 | 1.76E-20 |
| *RBM12B* | 1.774453 | 2.961842 | 0.73912 | 6.12E-18 | 4.05E-17 |
| *ESRP1* | 14.34576 | 25.65751 | 0.838757 | 8.83E-22 | 9.49E-21 |
| *RNH1* | 43.63035 | 23.83162 | -0.87246 | 7.67E-30 | 3.75E-28 |
| *RPP40* | 1.253982 | 3.242922 | 1.370778 | 2.93E-24 | 4.86E-23 |
| *SMG6* | 3.289602 | 2.154313 | -0.61069 | 2.57E-18 | 1.79E-17 |
| *LRRFIP1* | 29.68487 | 13.43099 | -1.14416 | 1.08E-31 | 1.19E-29 |
| *EIF4E3* | 6.794603 | 2.87743 | -1.23961 | 1.35E-30 | 8.90E-29 |
| *GTPBP1* | 12.66913 | 8.403038 | -0.59233 | 5.28E-21 | 5.05E-20 |
| *RPL36A* | 12.39232 | 18.05601 | 0.543033 | 4.11E-08 | 9.25E-08 |
| *MAGOHB* | 2.173482 | 3.536756 | 0.702418 | 7.00E-15 | 3.30E-14 |
| *IARS2* | 26.7133 | 39.32538 | 0.557903 | 1.33E-14 | 6.07E-14 |
| *EXO1* | 0.214572 | 2.379085 | 3.47087 | 1.03E-32 | 1.70E-30 |
| *DCP1A* | 8.392478 | 5.427658 | -0.62877 | 2.42E-24 | 4.09E-23 |
| *NANOS1* | 0.33635 | 0.904696 | 1.42747 | 5.80E-14 | 2.40E-13 |
| *PTRH2* | 2.886201 | 5.223912 | 0.855959 | 1.32E-21 | 1.37E-20 |
| *MRPS25* | 7.272133 | 11.11572 | 0.61215 | 5.66E-06 | 1.02E-05 |
| *RPP21* | 3.075794 | 4.74185 | 0.624491 | 4.70E-13 | 1.74E-12 |
| *IFIT2* | 15.1245 | 7.180587 | -1.07471 | 2.30E-20 | 1.96E-19 |
| *IFIT5* | 13.41986 | 8.509617 | -0.6572 | 9.97E-19 | 7.23E-18 |
| *IFIT1B* | 0.044831 | 0.020357 | -1.13896 | 1.13E-06 | 2.19E-06 |
| *IFIT3* | 38.53572 | 20.09942 | -0.93904 | 1.40E-20 | 1.23E-19 |
| *NOVA2* | 2.758095 | 0.490052 | -2.49266 | 1.14E-34 | 1.51E-31 |
| *PTCD1* | 0.971017 | 1.422568 | 0.55093 | 1.65E-10 | 4.75E-10 |
| *MRPS6* | 7.758457 | 11.11702 | 0.518929 | 5.68E-05 | 9.55E-05 |
| *SRRM4* | 0.048667 | 0.026417 | -0.88147 | 7.72E-25 | 1.52E-23 |
| *CNP* | 10.69439 | 15.2032 | 0.507521 | 6.60E-17 | 3.91E-16 |
| *LIN28A* | 0.003951 | 0.316423 | 6.32363 | 0.018229 | 0.022803 |
| *R3HCC1* | 15.20635 | 10.67957 | -0.50982 | 1.32E-17 | 8.56E-17 |
| *MARS* | 11.53051 | 17.16958 | 0.574399 | 5.51E-15 | 2.65E-14 |
| *DUS1L* | 9.612389 | 18.62387 | 0.954186 | 5.01E-24 | 7.63E-23 |
| *SETX* | 11.53539 | 8.012757 | -0.5257 | 5.22E-21 | 5.03E-20 |
| *MARS2* | 2.059535 | 3.448527 | 0.743661 | 5.26E-17 | 3.19E-16 |
| *MTIF2* | 7.717631 | 11.11345 | 0.526076 | 9.40E-24 | 1.36E-22 |
| *JAKMIP1* | 0.307937 | 0.678319 | 1.139327 | 5.59E-07 | 1.11E-06 |
| *FASTKD1* | 3.017689 | 4.410992 | 0.547659 | 1.17E-14 | 5.39E-14 |
