# Supplementary material for: Predicting the Clinical Outcome of Lung Adenocarcinoma Using a Novel Gene Pair Signature Related to RNA-Binding Protein
Source: Biomed Res Int. 2020 Oct 26;2020:8896511. doi: 10.1155/2020/8896511 (PMC7643376; doi:10.1155/2020/8896511)
Supplement: Supplementary 6 — Supplementary Table 2: results of KEGG enrichment analysis of genes in the signature. [file 8896511.f6.docx]

Table S2 Results of KEGG enrichment analysis of genes in the signature

| ID | Description | GeneRatio | BgRatio | P-value | P.adjust | Q-value | Gene symbol | Count |
| --- | --- | --- | --- | --- | --- | --- | --- | --- |
| hsa03018 | RNA degradation | 5/19 | 79/8040 | 8.41E-07 | 1.51E-05 | 1.06E-05 | SKIV2L/PABPC3/PABPC1L/DCP1A/DCPS | 5 |
| hsa03013 | RNA transport | 6/19 | 180/8040 | 2.47E-06 | 2.22E-05 | 1.56E-05 | MAGOHB/PABPC3/RAE1/PABPC1L/RPP40/POP7 | 6 |
| hsa03015 | mRNA surveillance pathway | 3/19 | 91/8040 | 0.001192 | 0.007152 | 0.005019 | MAGOHB/PABPC3/PABPC1L | 3 |
| hsa03040 | Spliceosome | 3/19 | 149/8040 | 0.004861 | 0.021876 | 0.015352 | SNRPA1/SLU7/MAGOHB | 3 |
| hsa00970 | Aminoacyl-tRNA biosynthesis | 2/19 | 66/8040 | 0.010373 | 0.037341 | 0.026204 | DARS2/PARS2 | 2 |
