# Supplementary material for: Predicting the Clinical Outcome of Lung Adenocarcinoma Using a Novel Gene Pair Signature Related to RNA-Binding Protein
Source: Biomed Res Int. 2020 Oct 26;2020:8896511. doi: 10.1155/2020/8896511 (PMC7643376; doi:10.1155/2020/8896511)
Supplement: Supplementary 7 — Supplementary Table 3: risk score-based GSEA for MSigDB oncogenic signature gene sets. [file 8896511.f7.docx]

Table S3 Risk score-based GSEA enrichment analysis for MSigDB oncogenic signatures gene sets

| Gene sets | Size | ES | NES | NOM p-val | FDR q-val | FWER p-val | RANK AT MAX | LEADING EDGE |
| --- | --- | --- | --- | --- | --- | --- | --- | --- |
| JAK2_DN.V1_DN | 143 | 0.529488 | 2.035148 | 0 | 0.060912 | 0.028 | 12831 | tags=51%, list=23%, signal=66% |
| BRCA1_DN.V1_DN | 136 | 0.381957 | 1.612404 | 0.001938 | 0.421664 | 0.435 | 13246 | tags=35%, list=24%, signal=45% |
| CSR_EARLY_UP.V1_DN | 125 | 0.467911 | 1.831006 | 0.003824 | 0.1542 | 0.14 | 14060 | tags=49%, list=25%, signal=65% |
| MTOR_UP.N4.V1_DN | 176 | 0.470522 | 1.852466 | 0.00396 | 0.19 | 0.121 | 12844 | tags=45%, list=23%, signal=59% |
| EGFR_UP.V1_DN | 179 | 0.391484 | 1.691574 | 0.010225 | 0.283149 | 0.31 | 14379 | tags=40%, list=26%, signal=53% |
| STK33_NOMO_DN | 271 | 0.3487 | 1.54912 | 0.01227 | 0.382589 | 0.521 | 17350 | tags=43%, list=31%, signal=62% |
| STK33_SKM_DN | 269 | 0.351518 | 1.584541 | 0.012371 | 0.338918 | 0.474 | 14590 | tags=37%, list=26%, signal=50% |
| PRC1_BMI_UP.V1_UP | 182 | 0.346742 | 1.543021 | 0.018987 | 0.362032 | 0.531 | 17384 | tags=45%, list=31%, signal=66% |
| MEK_UP.V1_DN | 179 | 0.425164 | 1.716175 | 0.022822 | 0.298906 | 0.281 | 16724 | tags=48%, list=30%, signal=69% |
| TBK1.DF_UP | 283 | 0.381687 | 1.608061 | 0.022857 | 0.326419 | 0.443 | 12485 | tags=35%, list=23%, signal=45% |
| E2F3_UP.V1_DN | 157 | 0.34332 | 1.510175 | 0.025918 | 0.309246 | 0.567 | 8455 | tags=25%, list=15%, signal=29% |
| YAP1_UP | 46 | 0.400993 | 1.52296 | 0.027505 | 0.323189 | 0.552 | 18212 | tags=48%, list=33%, signal=71% |
| DCA_UP.V1_DN | 180 | 0.363257 | 1.533177 | 0.02863 | 0.353553 | 0.542 | 18686 | tags=49%, list=34%, signal=74% |
| BCAT_BILD_ET_AL_DN | 45 | 0.484574 | 1.612403 | 0.034343 | 0.361426 | 0.435 | 12381 | tags=51%, list=22%, signal=66% |
| ESC_V6.5_UP_EARLY.V1_UP | 165 | 0.354625 | 1.469572 | 0.043478 | 0.316304 | 0.627 | 17014 | tags=42%, list=31%, signal=61% |
| STK33_DN | 270 | 0.319786 | 1.406234 | 0.047423 | 0.340346 | 0.702 | 17350 | tags=39%, list=31%, signal=57% |
| TGFB_UP.V1_DN | 186 | 0.331075 | 1.446353 | 0.049485 | 0.315766 | 0.658 | 14761 | tags=34%, list=27%, signal=47% |
| PRC2_EED_UP.V1_UP | 182 | 0.319202 | 1.415447 | 0.05071 | 0.337864 | 0.697 | 12451 | tags=34%, list=23%, signal=43% |
