# Supplementary material for: Predicting the Clinical Outcome of Lung Adenocarcinoma Using a Novel Gene Pair Signature Related to RNA-Binding Protein
Source: Biomed Res Int. 2020 Oct 26;2020:8896511. doi: 10.1155/2020/8896511 (PMC7643376; doi:10.1155/2020/8896511)
Supplement: Supplementary 8 — Supplementary Table 4: PPI network analysis between genes in the signature. [file 8896511.f8.docx]

Table S4 PPI network analysis between genes in the signature.

| Node1 | Node2 | Coexpression | Experimentally_determined_interaction | Database_annotated | Automated_textmining | Combined_score |
| --- | --- | --- | --- | --- | --- | --- |
| *WDR46* | *DCAF13* | 0.902 | 0.792 | 0.9 | 0.766 | 0.999 |
| *IGF2BP1* | *IGF2BP3* | 0.97 | 0.99 | 0 | 0.711 | 0.999 |
| *MRPS12* | *MRPL54* | 0.505 | 0.8 | 0.9 | 0.621 | 0.995 |
| *DDX52* | *DCAF13* | 0.858 | 0.302 | 0.9 | 0.616 | 0.995 |
| *SLU7* | *SNRPA1* | 0 | 0.926 | 0.9 | 0.245 | 0.993 |
| *MRPS12* | *MRPL38* | 0.143 | 0.846 | 0.9 | 0.451 | 0.991 |
| *DDX52* | *WDR46* | 0.901 | 0 | 0.9 | 0.221 | 0.991 |
| *MRPL54* | *MRPL38* | 0.135 | 0.8 | 0.9 | 0.577 | 0.991 |
| *MRPL54* | *MRPS24* | 0.459 | 0.8 | 0.9 | 0 | 0.988 |
| *DDX24* | *DDX56* | 0.892 | 0.834 | 0 | 0.793 | 0.988 |
| *MAGOHB* | *SNRPA1* | 0.221 | 0.8 | 0.9 | 0.234 | 0.986 |
| *MRPS12* | *MRPS24* | 0.241 | 0.824 | 0.9 | 0 | 0.985 |
| *RPP40* | *POP7* | 0.108 | 0.387 | 0.9 | 0.748 | 0.984 |
| *MRPS24* | *MRPL38* | 0.168 | 0.83 | 0.9 | 0 | 0.984 |
| *MAGOHB* | *SLU7* | 0.062 | 0.8 | 0.9 | 0.138 | 0.981 |
| *RAE1* | *MAGOHB* | 0.124 | 0 | 0.9 | 0.43 | 0.945 |
| *DDX52* | *DDX56* | 0.899 | 0.122 | 0 | 0.626 | 0.936 |
| *DDX24* | *DDX52* | 0.889 | 0.122 | 0 | 0.5 | 0.932 |
| *DCP1A* | *MAGOHB* | 0.068 | 0 | 0.9 | 0.3 | 0.929 |
| *WDR46* | *DDX56* | 0.906 | 0.14 | 0 | 0.172 | 0.927 |
| *SNRPA1* | *PPIL4* | 0.102 | 0.182 | 0.9 | 0 | 0.92 |
| *DDX24* | *URB1* | 0.84 | 0.392 | 0 | 0.226 | 0.918 |
| *URB1* | *DDX56* | 0.838 | 0.157 | 0 | 0.434 | 0.916 |
| *RPP40* | *DCAF13* | 0.143 | 0 | 0.9 | 0 | 0.91 |
| *RPP40* | *WDR46* | 0.105 | 0 | 0.9 | 0 | 0.906 |
| *DDX24* | *WDR46* | 0.886 | 0.14 | 0 | 0.111 | 0.905 |
| *SLU7* | *PPIL4* | 0.063 | 0 | 0.9 | 0.063 | 0.904 |
| *MAGOHB* | *PPIL4* | 0.076 | 0 | 0.9 | 0 | 0.903 |
| *DDX52* | *RPP40* | 0.072 | 0 | 0.9 | 0 | 0.903 |
| *INTS8* | *ZC3H8* | 0.063 | 0 | 0.9 | 0 | 0.902 |
| *RAE1* | *SLU7* | 0 | 0 | 0.9 | 0.057 | 0.901 |
| *DCAF13* | *DDX56* | 0.879 | 0.053 | 0 | 0.056 | 0.882 |
| *RNPC3* | *SNRPA1* | 0.13 | 0.695 | 0 | 0.537 | 0.866 |
| *DDX24* | *DCAF13* | 0.845 | 0.053 | 0 | 0.056 | 0.849 |
| *WDR4* | *DDX56* | 0.847 | 0 | 0 | 0 | 0.847 |
| *DDX52* | *URB1* | 0.782 | 0 | 0 | 0.204 | 0.819 |
| *OAS1* | *OASL* | 0.785 | 0 | 0 | 0.755 | 0.81 |
| *TFB2M* | *DCAF13* | 0.797 | 0 | 0 | 0.094 | 0.808 |
| *WDR4* | *WDR46* | 0.687 | 0 | 0 | 0.352 | 0.788 |
| *DDX52* | *TFB2M* | 0.756 | 0 | 0 | 0.097 | 0.77 |
| *TFB2M* | *DDX56* | 0.732 | 0.064 | 0 | 0.086 | 0.753 |
| *WDR46* | *TFB2M* | 0.684 | 0.05 | 0 | 0.21 | 0.742 |
| *DDX24* | *TFB2M* | 0.714 | 0.064 | 0 | 0.086 | 0.736 |
| *PARS2* | *DARS2* | 0.225 | 0 | 0 | 0.608 | 0.728 |
| *SPATS2* | *STRBP* | 0.072 | 0 | 0 | 0.697 | 0.706 |
| *ZC3H12D* | *ZC3HAV1L* | 0 | 0 | 0 | 0.657 | 0.657 |
| *DDX24* | *WDR4* | 0.64 | 0 | 0 | 0 | 0.64 |
| *DDX52* | *PARS2* | 0.089 | 0 | 0 | 0.61 | 0.63 |
| *MRPS12* | *WDR46* | 0.113 | 0.567 | 0 | 0 | 0.599 |
| *MRPS12* | *DCAF13* | 0.088 | 0.567 | 0 | 0 | 0.588 |
| *RPP40* | *TSEN54* | 0 | 0 | 0.54 | 0.133 | 0.584 |
| *URB1* | *WDR46* | 0.58 | 0 | 0 | 0 | 0.58 |
| *DCP1A* | *DCPS* | 0.121 | 0 | 0 | 0.532 | 0.571 |
| *DDX52* | *SKIV2L* | 0.19 | 0 | 0 | 0.49 | 0.569 |
| *ZC3H12C* | *ZC3HAV1L* | 0 | 0 | 0 | 0.567 | 0.567 |
| *SKIV2L* | *DCPS* | 0.067 | 0 | 0 | 0.532 | 0.545 |
| *URB1* | *DCAF13* | 0.543 | 0 | 0 | 0 | 0.543 |
| *TSEN54* | *POP7* | 0 | 0 | 0.54 | 0 | 0.54 |
| *MRPS12* | *TFB2M* | 0.1 | 0.375 | 0 | 0.199 | 0.531 |
| *DDX24* | *SKIV2L* | 0.065 | 0.313 | 0 | 0.282 | 0.498 |
| *PABPC1L* | *SNRPA1* | 0 | 0.282 | 0 | 0.308 | 0.481 |
| *MRPS12* | *DARS2* | 0.122 | 0 | 0 | 0.273 | 0.478 |
| *SKIV2L* | *PPIL4* | 0.121 | 0 | 0 | 0.424 | 0.473 |
| *ERI2* | *SNRPA1* | 0.062 | 0 | 0 | 0.447 | 0.459 |
| *DCP1A* | *SKIV2L* | 0.064 | 0.266 | 0 | 0.269 | 0.454 |
| *DNMT3B* | *IGF2BP1* | 0.441 | 0 | 0 | 0.046 | 0.443 |
| *DDX52* | *WDR4* | 0.425 | 0 | 0 | 0 | 0.424 |
| *URB1* | *TFB2M* | 0.421 | 0 | 0 | 0 | 0.42 |
| *RNPC3* | *SLU7* | 0.062 | 0.341 | 0 | 0.116 | 0.405 |
